# Virtual Screening and the In Vitro Assessment of the Antileishmanial Activity of Lignans

**DOI:** 10.3390/molecules25102281

**Published:** 2020-05-12

**Authors:** Mayara dos Santos Maia, Joanda Paolla Raimundo e Silva, Thaís Amanda de Lima Nunes, Julyanne Maria Saraiva de Sousa, Gabriela Cristina Soares Rodrigues, Alex France Messias Monteiro, Josean Fechine Tavares, Klinger Antonio da Franca Rodrigues, Francisco Jaime B. Mendonça-Junior, Luciana Scotti, Marcus Tullius Scotti

**Affiliations:** 1 Laboratory of Cheminformatics, Program of Natural and Synthetic Bioactive Products (PgPNSB), Health Sciences Center, Federal University of Paraíba, João Pessoa-PB 58051-900, Brazil; mayarasmaia@hotmail.com (M.d.S.M.); gaby.ecologia@gnail.com (G.C.S.R.); alexfrancem@ltf.ufpb.br (A.F.M.M.); luciana.scotti@gmail.com (L.S.); 2 Multi-User Characterization and Analysis Laboratory, Program of Natural and Synthetic Bioactive Products (PgPNSB), Health Sciences Center, Federal University of Paraíba, João Pessoa-PB 58051-900, Brazil; joanda@ltf.ufpb.br (J.P.R.e.S.); josean@ltf.ufpb.br (J.F.T.); 3 Infectious Diseases Laboratory, Federal University of Parnaíba Delta, São Benedito, Parnaíba-PI 64202-020, Brazil; thaisaln13@gmail.com (T.A.d.L.N.); jully.yanne@gmail.com (J.M.S.d.S.); klinger.antonio@gmail.com (K.A.d.F.R.); 4 Laboratory of Synthesis and Drug Delivery, State University of Paraíba, João Pessoa-PB 58071-160, Brazil; franciscojbmendonca@yahoo.com.br

**Keywords:** leishmaniasis, lignan, virtual screening, molecular docking, computer-aided drug design

## Abstract

Leishmaniasis is endemic in at least 98 countries. Due to the high toxicity and resistance associated with the drugs, we chose lignans as an alternative, due to their favorable properties of absorption, distribution, metabolism, excretion, and toxicity (ADMET). To investigate their leishmanicidal potential, the biological activities of a set of 160 lignans were predicted using predictive models that were built using data for *Leishmania* *major* and *L. (Viannia) braziliensis*. A combined analysis, based on ligand and structure, and several other computational approaches were used. The results showed that the combined analysis was able to select 11 lignans with potential activity against *L. major* and 21 lignans against *L. braziliensis*, with multitargeting effects and low or no toxicity. Of these compounds, four were isolated from the species *Justicia* *aequilabris* (Nees) Lindau. All of the identified compounds were able to inhibit the growth of *L. braziliensis* promastigotes, with the most active compound, (**159**) epipinoresinol-4-*O*-β-d-glucopyranoside, presenting an IC_50_ value of 5.39 µM and IC_50_ value of 36.51 µM for *L. major*. Our findings indicated the potential of computer-aided drug design and development and demonstrated that lignans represent promising prototype compounds for the development of multitarget drugs against leishmaniasis.

## 1. Introduction

Leishmaniasis refers to a collection of diseases, caused by intracellular parasites of the *Leishmania* genus [1]. Transmission can occur in humans through the bite of Diptera, in the Psychodida family, which are hematophagous insects in the genera *Phlebotomus* or *Lutzomyia* [2]. Leishmaniasis is endemic in at least 98 countries [3] and is considered to be a neglected disease because it affects primarily low-income populations but remains a relevant public health problem, with considerable incidence and prevalence rates in Brazil [4]. The World Health Organization considers leishmaniasis to be one of the six most neglected tropical diseases, affecting 2 million people annually, worldwide [5]. More than 20,000 deaths are estimated to occur annually, due to disease complications [6].

According to Akhoundi et al., 21 species of the genus Leishmania are known to be pathogenic to humans, including *L. major*, *L. tropica*, *L. aethiopica*, *L. donovani*, *L. infantum*, *L. mexicana*, *L. amazonensis*, *L. braziliensis*, *L. peruviana*, *L. guyanensis*, *L. panamemsis*, *L shawi*, *L. lainsoni*, *L. lindenbergi*, *L. martiniquensis*, *L. siamensis*, and *L. colombiensis*. [7]. Leishmaniasis can be categorized as tegumental and visceral leishmaniasis, and both can be further subdivided into other clinical forms of infection, defined by the location of the parasite in infected tissues and by the species, including cutaneous leishmaniasis (CL), mucocutaneous leishmaniasis (MCL), visceral or kala azalis leishmaniasis (VL), and post-kala azal dermal leishmaniasis (PKDL) [8]. The most common form is CL, and more than 90% of all CL cases are distributed across three primary regions: (i) Afghanistan, Iran, Saudi Arabia, and Syria; ii) Algeria and Tunisia; and (iii) Brazil and Peru [9].

CL symptoms present as one or more lesions, which are often painless, and purulence is uncommon. Lesions are typically located in exposed areas, such as the face and extremities, with ulcers and nodule/plaque appearances being common, and transmission occurs through the bites of mosquitos infected with the parasite [10]. Although not fatal, multiple lesions tend to leave permanent scars that can lead to social stigmatization.

In the Americas, the primary etiological agent of CL is *L. braziliensis* [11], which generally encompasses severe clinical forms associated with skin, mucosal, mucocutaneous, and subcutaneous nodular lesions [12,13], and large genetic polymorphisms can cause visible deformities in the host, in addition to psychological, social, and economic impacts [14]. CL is a chronic inflammatory disease that is widely distributed in Brazil and primarily caused by *L. braziliensis* [15].

In India, the Middle East, Central Asia, and North and West Africa, CL is primarily caused by the species *L. major* [12,16], causing mild to severe skin disorders that can result in disfigurement if left untreated [17]. In the Middle Eastern region and Israel, CL is frequently caused in humans by infection through wild mammal reservoirs, with *Phlebotomus papatasi* being the primary vector for *L. major* [16]. Although *Leishmania* parasites affect millions of people, in several countries, around the world, no human vaccine is currently available for the treatment of CL caused by *L. major*, and the available treatments are expensive and have toxic side-effects. Thus, the identification of new therapeutic options against the disease remains necessary [18].

Current treatments for leishmaniasis are based on chemotherapeutic drugs, which are often inefficient or harmful, due to the development of drug resistance and side effects, associated with the high toxicity index. In addition, no vaccines are currently available, although some vaccine candidates for the treatment of these diseases are currently in the pre-clinical and clinical testing phases [19,20].

Because *L. major* and *L. braziliensis* cause lesions in the host that can be disseminated to other sites and the exacerbated production of cytokines and chemokines that cause oxidative stress, trigger the amplification of the inflammatory response [21], we chose liganans and neolignans because they have properties favorable to drug development. Factors in the host such as immunosuppressant, malnutrition, and co-infection or genetic and environmental factors are factors that aggravate the disease. In this sense, we chose lignans and neolignans to investigate antileishmania activity in enzymes important for the survival and proliferation of parasites, reducing injuries and decrease the inflammatory response [22]. In addition, lignans are known for their anti-inflammatory and antioxidant activity, which could minimize the effects of the inflammatory response. In addition, a study by Pilkington [23], evaluating the absorption, distribution, metabolism, excretion, and toxicity (ADMET) profiles of lignans found that more than 75% of lignans met all the requirements for drug-likeness. The study concluded that lignans show a high level of drug similarity.

Computational tools can contribute greatly to database creation, by predicting protein functions, modeling protein structures, simulating metabolic pathway kinetics, predicting biological activities, predicting toxicity, and predicting the affinities and flexibilities between receptors and ligands, which can facilitate the development and identification of drugs with the potential to treat various diseases and promote the development of efficacious drugs with reduced toxicity [24,25].

Therefore, this study aimed to use virtual screening and experimental validation to identify lignans with leishmanicidal potential, low toxicity, and selective activity against several Leishmania targets.

## 2. Results

### 2.1. Prediction of ADMET Properties

Various predictive parameters were determined for a set of 160 lignans and neolignans, to identify compounds with the best ADMET profiles for further examination using other methodologies. The results showed that among the 160 lignans and neolignanas, only 34 failed the Lipinski rule. Because the application of the Lipinski rule did not decisively filter the molecules, we used additional methodologies to select those compounds with the best profiles.

During the analysis of lipophilicity and water solubility, 148 compounds (92%) obtained good results, presenting consensus log *p* values below <4.15 and/or at least two descriptors with the classification “Low solubility” (Table S1). Then, the 148 compounds were submitted to pharmacokinetic analyzes. The results showed that 42 lignans (28.3%) had adequate pharmacokinetics (Table S1).

Toxicity was assessed for the 42 lignans and we found that 33 (78%) of the 42 compounds with good pharmacokinetic action had low or no predicted risk for the development of mutagenicity, tumorigenesis, negative effects on the reproductive system, or irritability (Table S2).

### 2.2. Quantitative Structure-Activity Relationship (QSAR) Modeling

To perform ligand-based virtual screening, two prediction models were built, using the random forest (RF) algorithm. To construct these models, molecular descriptors were calculated for the bank of molecules with known activity against *L. major* and *L. braziliensis*, obtained through the ChEMBL database. After validating the models, 33 lignans with excellent ADMET profiles were analyzed for leishmanicidal activity, using the prediction models.

**Figure 1:**
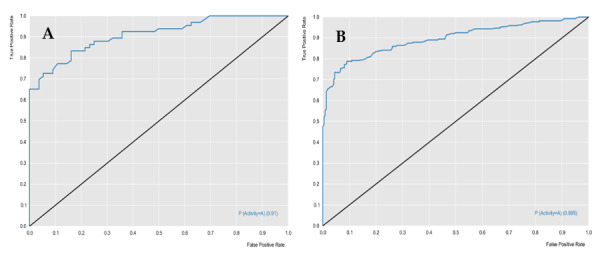
Receiver operating characteristic (ROC) curve generated for the *L. major* random forest (RF) model. (**A**) Test and (**B**) cross-validation.

**Figure 2:**
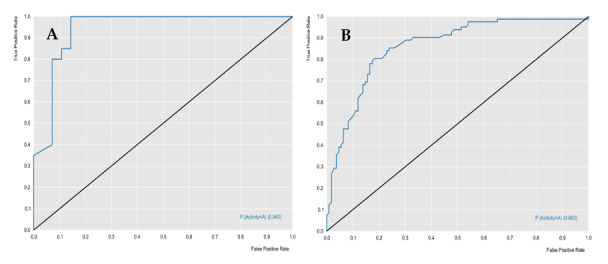
ROC curve generated for the *L. braziliensis* RF model. (**A**) Test and (**B**) cross-validation.

The RF models were evaluated for their predictive powers, using the parameters of specificity, sensitivity, accuracy, positive predicted value (PPV), and negative predicted value (NPV), in addition to performance and robustness, such as the receiver operating characteristic (ROC) curve and Mathews correlation coefficient (MCC). Table 1 describes the characteristics of the two models, in terms of predictive power and robustness, and Figure 1 and Figure 2 show the performances of the models.

The cross-validation results demonstrated that the generated models obtained excellent performance results, with an accuracy greater than 76%. The *L. major* ROC curve showed a value greater than 0.89 and the MCC value was 0.63, which indicated that the model demonstrated excellent classification, performance, and robustness rates. The ROC curve for the *L. braziliensis* model was greater than 0.86, with an MCC value equal to 0.87, which were also good results.

With the models created and demonstrated to have excellent performance, the lignan bank was then screened to select compounds that are potentially active against *L. major* and *L. braziliensis*.

The RF model was able to select 11 compounds with active potential, with probabilities ranging from 50% to 57%, for *L. major* (Table 2), whereas the model for *L. braziliensis* was able to select 21 potentially active compounds, with probabilities ranging between 50% and 75% (Table 3). Compounds 86 and 160 were considered to be most active for *L. major*, and compounds 8, 60, 157, and 160 were considered to be the most active for *L. braziliensis*.

### 2.3. Alignment of Protein Sequences

Sequence alignment was used to verify the similarities and identities of the enzymes selected in this study, across different species, which allowed the analysis of conserved regions and the identification of common residues in the active site. In addition, differences and structural similarities could be identified that might contribute to rational drug planning. Therefore, we investigated the shared amino acids in the active sites of various *L. major* and *L. braziliensis* enzymes.

The results showed that most of the enzymes shared greater than 80% identity between *L. major* and *L. braziliensis*, with 84% identity for Glycerol-3-phosphate dehydrogenase (GPDH); 85% for dihydroorotate dehydrogenase (DHODH); 72.13% for the Pteridine reductase 1 (PTR1); 82.69% for Trypanothione reductase (TR); and 85.69% for UDP-glucose pyrophosphorylase (UGPase) (Figures S1–S5).

All of the amino acids in the active site of GPDH protein were conserved between species. According to Choe et al. [26], interactions with the residues Trp44, Ile93, Phe101, Phe97, and Met46 of *L. major* GPDH (*Lm*GPDH) were observed for the compound 2-bromo-6-chloro-purine. For the DHODH protein, researchers have described advances and perspectives in structural biology, which were used to identify and validate target sites for the development of drugs that target this enzyme [27]. The study showed that DHODH is structurally conserved among *Leishmania* species, except in *L. braziliensis*, which showed changes in the amino acid sequence of the active site. In *L. braziliensis*, Met104 is replaced with Ala138, and Cys150 is replaced with Tyr176 (Figure S2). In the PTR1 enzyme, interactions were observed between the amino acids Arg17, Ser111, Asp181, Leu188, Leu226, Leu229, His241, Tyr283, and Arg287 and the compound (2~{*R*})-2-[3,4-bis (oxidanyl) phenyl]-6-oxidanyl-2,3-dihydrochromen-4-one, indicating the location of the active site, according to a study performed by Pisa et al. [28]. In this study, we noticed the replacement of the Leu229 residue with Phe229 in the *L. braziliensis* PTR1. The enzymes TR and UGPase showed that the residues in the active site were highly conserved in both species.

### 2.4. Homology Modeling

In this study, six models were generated: *L. major* GPDH (*Lm*GPDH) *L. braziliensis* GPDH (*Lb*GDPH), *L. braziliensis* PTR1 (*Lb*PTR1), *L. major* TR *(Lm*TR), *L. braziliensis* TR (*Lb*TR), and *L. braziliensis* UGPase *(Lb*UGPase).

**Figure 3:**
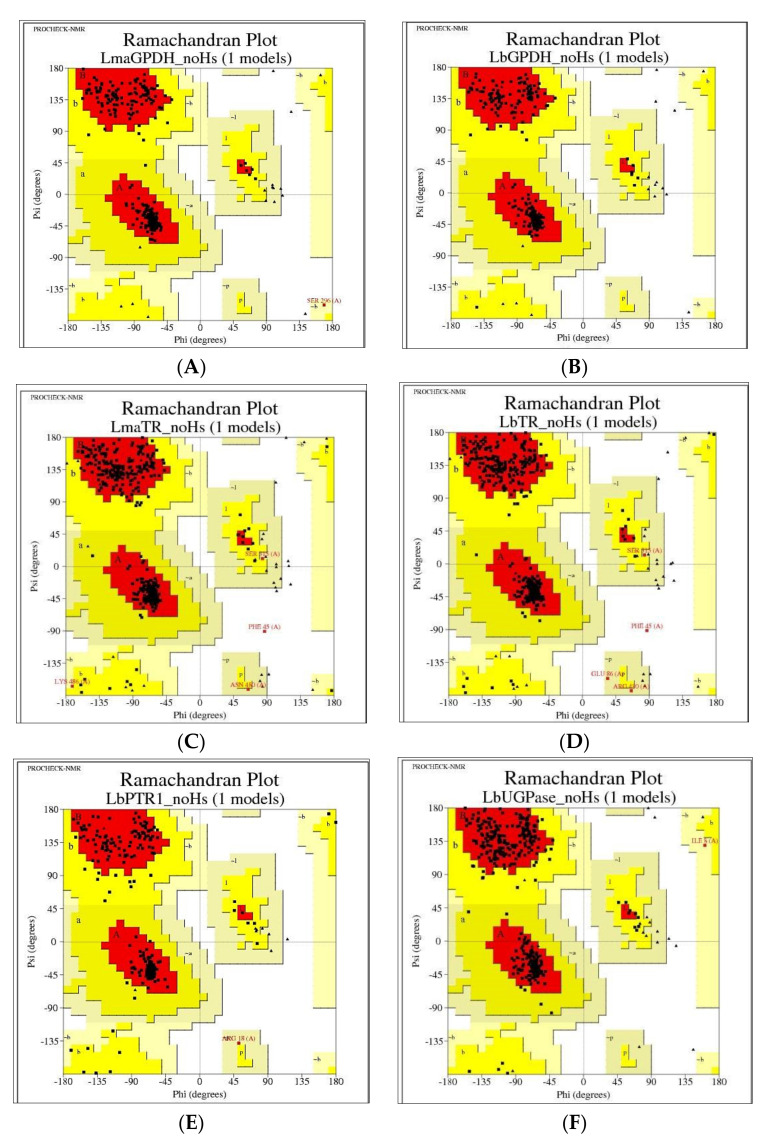
Ramachandran graphs of the homology models generated for *L. major* and *L. braziliensis* enzyme. The colored regions represent the permitted and favored regions of the secondary structures, and the white regions represent the prohibited regions. (**A**) Glycerol-3-phosphate dehydrogenase (GPDH) in *L. major*. (**B**) GPDH in *L. braziliensis*. (**C**) Trypanothione reductase (TR) in *L. major*. (**D**) TR in *L. braziliensis*. (**E**) Pteridine reductase 1 (PTR1) in *L. braziliensis.* (**F**) UDP-glucose pyrophosphorylase (UGPase) in *L. braziliensis*.

The reliabilities of the models were verified using several tools. One of the primary tools used was the Ramachandran graph, which represents all possible combinations of dihedral angles Ψ (psi) versus φ (phi) for each amino acid of a protein, except for glycine, which has no side chains, and models are considered to be reliable when more than 90% of amino acids are present in the permitted and/or favored regions (colored regions of the graph). Blank regions represent outliers, which have bad contacts. All generated models showed greater than 98% of amino acids in the permitted and favored regions (Figure 3 and Table 4). Highly reliable models were likely obtained due to the high similarity between the model sequences and the high resolution of the templates. All models were used for the following methodologies.

Verify 3D analyzes the compatibility of the 3-dimensional (3D) structure with its 1-dimensional (1D) amino acid sequence, based on the characteristics of the chemical environment, such as polarity and compares the results with good structures.

The method determines the environmental characteristics of each residue: (i) The total area of the side chain; ii) the fraction of the side chain area covered by polar atoms or water; and (iii) the local secondary structure. Then, the information is categorized and the structure of the 3D protein is converted into 1D, as a sequence, which represents the environment class of each residue in the structure of the folded protein. Then, the sequence is aligned and compared with sequences of good structures. A reliable model must have a 3D-1D score of more than 80%. All models obtained had scores above 80%, as shown in Table 5.

The quality of the atomic contacts between the atoms of each residue was analyzed, using the module Coarse Packing Quality Control or Fine Packing Quality Control on the WHAT IF server, which compares the distribution of atom positions around each residue. A mean score of less than –5.0 indicates bad or unusual atomic contacts. All models generated by the homology presented average score values above −5.0, as shown in Table 6.

### 2.5. Combined Analysis, Based on Ligand and Structure

The lignans and neolignans that were considered to be active based on the RF models for *L. major* and *L. braziliensis* were submitted to docking consensus evaluation, to increase the reliability of the method and decrease the number of false positives.

In total, 10 enzymes were used, four obtained from the PDB database and six based on homology. The docking results were generated using five different scoring functions and were validated by redocking the PDB ligand with the five types of enzymes for each species. More negative values indicated better predictions for most scoring function, except for the Goldscore and ChemPLP, which rank the best poses using the most positive values.

After docking, the results were standardized, and an average of all energy values was calculated for each lignan. Then, the lignans that obtained lower energy values than the PDB ligand in at least three of the scoring functions were used.

Further calculations were performed to obtain the lignans with the best active potential probabilities for each of the analyzed proteins, using the following formula:

(1) $$Prob=\frac{EM_{Lig}}{EM_{MLig}},\;se\;EM_{Lig}<EM_{Inib}$$

where *EM_Lig_* is the average energy across all five scoring functions for each analyzed lignan, *EM_MLig_* is the highest average energy obtained by the tested lignans, and *EM_Inib_* is the average energy across all five scoring functions for the ligand inhibitor, obtained from the crystallographic data of the test protein. Thus, only those compounds that obtained energy values equal to or greater than the interaction energy of the crystallographic inhibitor ligand were considered to be potentially active.

Among the 11 lignans analyzed for *L. major*, all were considered potentially active against GPDH, 10 against DHODH, 9 against PTR1, 9 against TR, and all 11 against UGPase (Table S3).

Among the 21 lignans analyzed using the consensus docking analysis for *L. braziliensis*, 20 were potentially active against GPDH, all against DHODH, 19 against PTR1, 16 against TR, and 15 against UGPase (Table S4).

Tables S3 and S4 show that the consensus docking analysis returned activity probabilities for the examined lignans that were much higher than that for PDB ligand for various enzymes in the two species.

A second consensus analysis was performed to select multitarget lignans, which demonstrate an active potential probability for more than one protein, in the RF analysis and. For this analysis, the following formula was used:

(2) $$Prob_{Comb}=\frac{(Prob_{Dc}+\left(1+ESP\right)\times P_{Activity}}{2+ESP},\;Se\;Prob_{Comb}>0.5$$

where *Prob_Dc_* is the average active potential probability based on the molecular coupling analysis, *ESP* is the specificity value from the RF model, and *P_Activity_* is the active potential probability value from the RF model. The combined probability is conditioned, such that only compounds with values above 0.5 are considered likely to be active. The combined probability values were calculated for the lignans for each enzyme studied. Finally, we analyzed which compounds were multitarget compounds. Table 7 and Table 8 show the combined probability values between the forecasting models and the molecular docking analysis.

**Figure 4:**
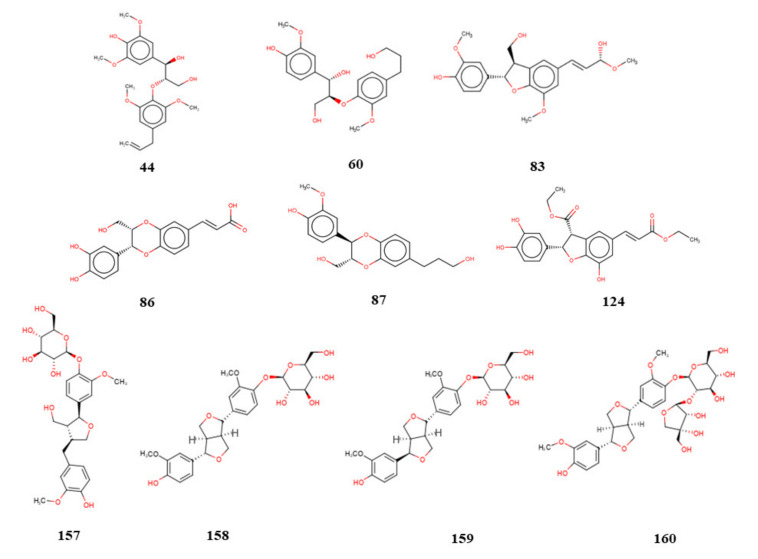
Common compounds that are considered to be potentially active against *L. major* and *L. braziliensis*, based on the random forest model, selected by the consensus analysis, combined with the fit values, and identified as multitarget.

Among the lignin and neolignans bank analyzed in this study, after the combined ligand- and structure-based analysis, and the identification of multitarget compounds, all of the lignans, except lignan 76, were considered to be potentially active against three or more enzymes (Table 7 and Table 8). Therefore, 20 molecules were considered to have potential multitarget activity against *L. major* and/or L*. braziliensis*, with 10 molecules common to both species. We also observed that the most likely targets, according to the combined analysis based on structure and ligand, are GPDH, PTR1, TR, and UGPase for *L. major*. While for *L. braziliensis*, the most likely targets are GPDH, PTR1, and UGPase. Figure 4 shows the common lignans for *L. major* and *L. braziliensis* that were considered to be potentially active, based on the RF model, selected by the consensus analysis combined with fit values, and identified as multitarget. We also observed that *L. braziliensis* showed higher rates of combined probability, indicating that lignans may be potential leishmanicides for this species.

#### 2.5.1. Interaction Analysis

**Figure 5:**
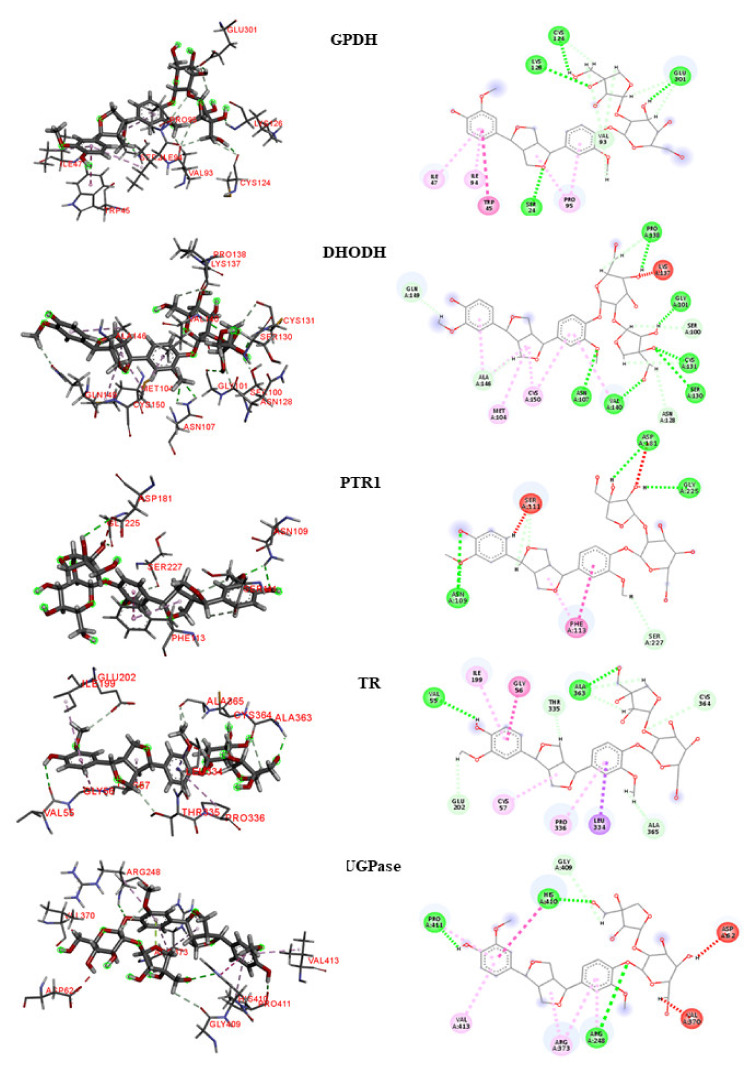
3D and 2D interactions between lignan 160 and the five examined enzymes [Glycerol-3-phosphate Dehydrogenase (GPDH), Dihydroorotate dehydrogenase (DHODH), Pteridine reductase 1 (PTR1), Trypanothione reductase (TR), and UDP—glucose pyrophosphorylase (UGPase)] in *L. major*. Hydrogen bonds are highlighted in green; hydrophobic interactions are highlighted in pink, and electrostatic interactions are highlighted in red.

**Figure 6:**
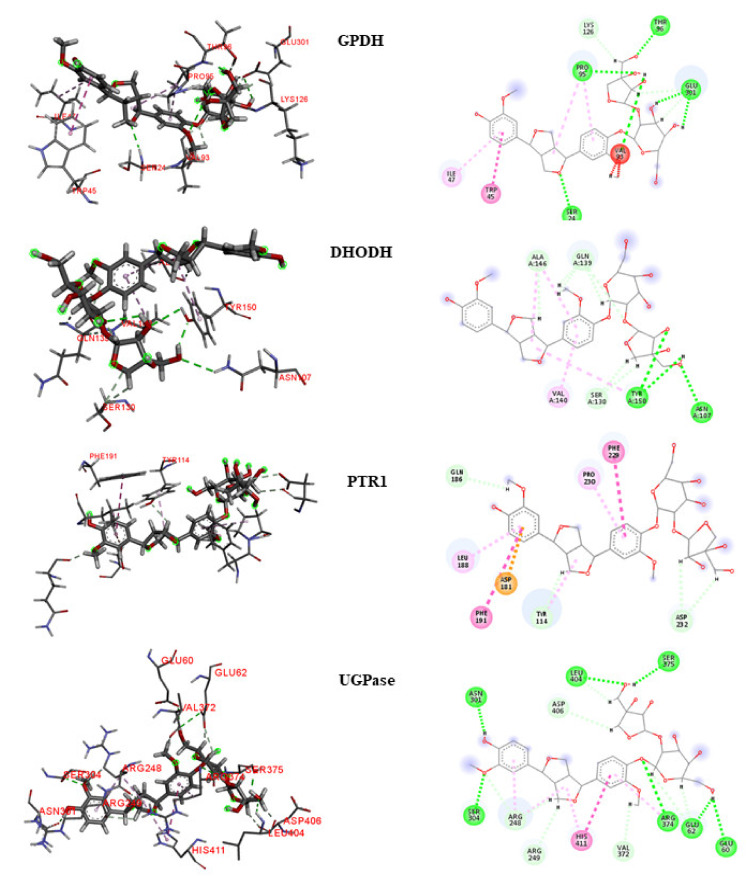
3D and 2D interactions between lignan 160 and four enzymes [Glycerol-3-phosphate Dehydrogenase (GPDH), Dihydroorotate dehydrogenase (DHODH), Pteridine reductase 1 (PTR1), and UDP—Glucose pyrophosphorylase (UGPase)] in *L. braziliensis*. Hydrogen bonds are highlighted in green, hydrophobic interactions are highlighted in pink, and electrostatic interactions are highlighted in red.

To analyze the interactions, we selected lignan **160** as the compound with the highest leishmanicidal potential because it is considered to be multitarget and obtained activity predictions for Leishmania species (Figure 5 and Figure 6).

##### GPDH

For the *L. major* GPDH enzyme, the interaction with lignan **160** consisted primarily of large numbers of hydrophobic bonds. Four hydrogen bonds were established, which consisted of bonds with the Ser24, Cys124, Lys126, and Glu301 residues. Four hydrophobic interactions were counted, corresponding with the Thr45, Ile47, Ile94, and Pro95 residues. We observed that although the lignan was located at the enzyme active site, it interacted with other amino acids that are not reported in the literature.

Similar to *L. major*, the *L. braziliensis* GDPH formed four hydrogen bonds with lignan **160**, at the Ser24, Pro95, Thr96, and Glu301 residues. Two hydrophobic interactions, with Trp45 and Ile47, and a steric Val93 interaction were also observed.

##### DHODH

In *L. major*, lignan 160 formed several hydrogen bonds with the DHODH enzyme active site, providing increased stability to the complex. Links with the amino acids Gly101, Asn107, Ser130, Cys131, Ser130, Pro138, and Val140 and hydrophobic interactions with the amino acids Met104 and Cys150 were observed. According to the literature, lignan **160** interacted with two important amino acids responsible for catalytic activity: Met104 and Cys150.

In *L. braziliensis*, two hydrogen bonds were formed with residues Asn107 and Tyr150, and three hydrophobic interactions were observed with Ser130, Gln139, and Ala146.

##### PTR1

Hydrogen bonds with the amino acids Ans109, Asn181, and Gly225 were observed between lignan **160** and the active site of *L. major* PTR1. A hydrophobic interaction with Phe113 and a steric interaction with Ser111 were also observed. Important interactions have been reported in the literature between the amino acids Arg17, Ser111, Asp181, Leu188, Leu226, Leu229, His241, Tyr283, and Arg287 and the compound (2~{*R*})-2-[3,4-bis(oxidanyl) phenyl ] -6-oxidanil-2,3-dihydrochromen-4-one, indicating that this may be the location of the active site, as reported in a study by Pisa et al. [25]. In the present study, we noticed the substitution of Leu229 residue with Phe229 in *L. braziliensis* PTR1, and a hydrophobic interaction was observed at Phe229. In addition, three hydrophobic interactions, with the amino acids Leu188, Phe191, and Pro230, were also observed. According to the literature [28], the Leu188 residue is conserved in Leishmania species.

##### TR

The amino acids in the TR active site formed several bonds with lignan 160. Hydrogen bonds were formed with the residues Val55 and Ala363 and hydrophobic interactions were observed with the residues Cys57, Ile199, and Pro336. Interactions with the amino acids Cys57 and Pro336 are considered important for enzymatic inhibition. In contrast, lignan **160** did not obtain good results in *L. braziliensis*.

##### UGPase

In *L. major*, lignan **160** established hydrogen bonds with the amino acids Arg248, His410, and Pro411. In addition, hydrophobic interactions with residues Arg373 and Val413 and steric interactions with the amino acids Asp62 and Val370 were observed. Most of these amino acids, such as Arg248, Arg373, His410, and Pro411, have been reported in the literature as important for protein inhibition.

In *L. braziliensis*, seven hydrogen bonds were observed between lignan 160 and the UGPase active site, providing increased stability. Links with the amino acids Glu60, Glu62, Ans301, Ser304, Arg374, Ser375, and Leu404 were observed, in addition to a hydrophobic interaction with His411.

#### 2.5.2. Docking Validation

The docking results generated by the five scoring functions were validated by redocking the PDB ligand with the five proteins from *Leishmania* species.

The root-mean-square deviations (RMSDs) of the obtained fitting poses were calculated in comparison with the crystal structure. RMSD values of less than 2 Å indicate an ideal degree of screening reliability. Information regarding the starting structures and the redocking validation results are shown in Table 9.

During the redocking analysis, most of the RMSD values were below 2.0 Å, and all five tested scoring functions positioned the ligand correctly at the active site. The Vina program generated only one ideal RMSD value. Thus, studies that use only the Vina program may generate many false-positive results. In addition, RMSD values for the poses obtained by AD4 could not be calculated because the program does not generate outputs for all ligand poses. Overall, the programs provided values that were considered to be satisfactory for the docking consensus validation.

#### 2.5.3. Evaluation of Docking Programs

**Figure 7:**
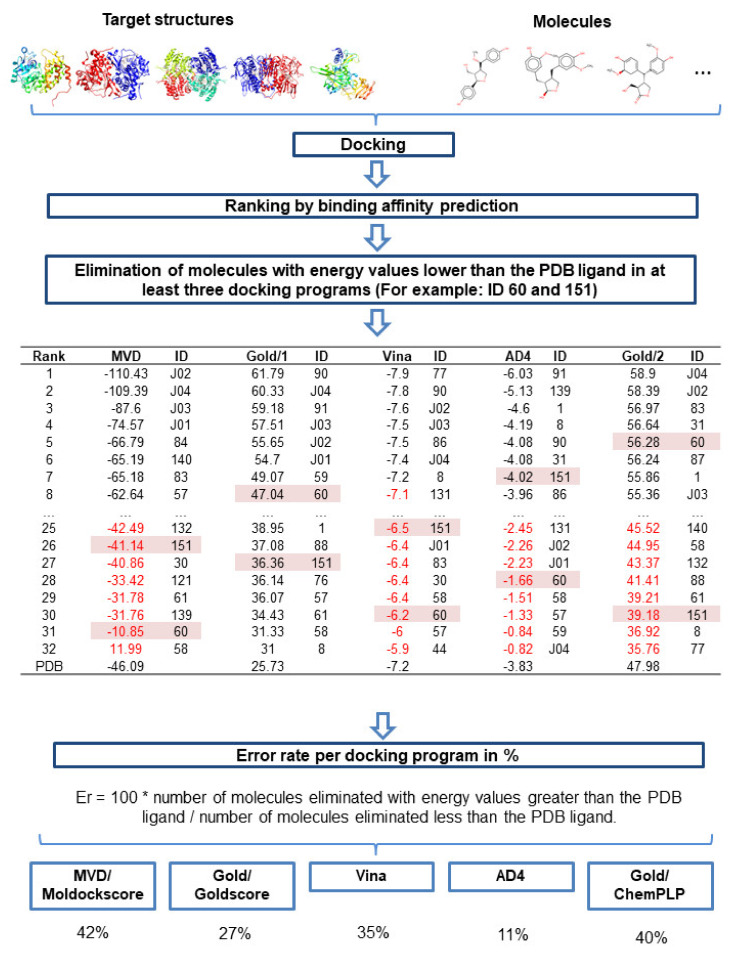
Workflow used to verify the performance of the docking programs, using the connection energy values.

The performances of the programs used for docking analysis in this study were evaluated, by analyzing how each program ranked the compounds that were discarded from the study for having lower energy values than the PDB ligand in at least three docking programs. The workflow used to perform the step-by-step calculations of program error rates (E_r_) is shown in Figure 7. The calculation of E_r_ was used to analyze the probability that a given program would classify an inactive molecule as active, verifying its performance.

The results showed that the MVD, Gold, Vina, AD4, and Plants programs presented E_r_ values of 42%, 27%, 35%, 11%, and 40%, respectively. Therefore, AD4 had the highest hit rate (89%). Although AD4 is more restrictive for the selection of active compounds, the program was able to obtain excellent results when compared against other programs. Table 10 shows the E_r_ value *per* program/enzyme, revealing that MVD had a higher total E_r_, with higher E_r_ values identified for DHODH and UGPase compared with those for the other programs.

### 2.6. Prediction of Drug Resistance

One of the great justifications for the development of new drugs against Leishmaniasis is the resistance of some species to commercialized drugs. To minimize the possible effects of resistance to a likely drug candidate, we searched the TritryDB database for single nucleotide polymorphisms (SNP). SNPs are mutations that are frequently present in over 1% of the species and may contribute to the development of drug resistance.

The results showed that only one SNP was identified in *L. major* PTR1, whereas in *L. braziliensis*, non-synonymous SNPs were identified in both DHODH and TR. No data were found for UGPase in either species.

Among the 14 SNPs identified, only four presented a polymorphic allele with relevant allele frequency, between 40% and 50%, as shown in Table 11.

**Figure 8:**
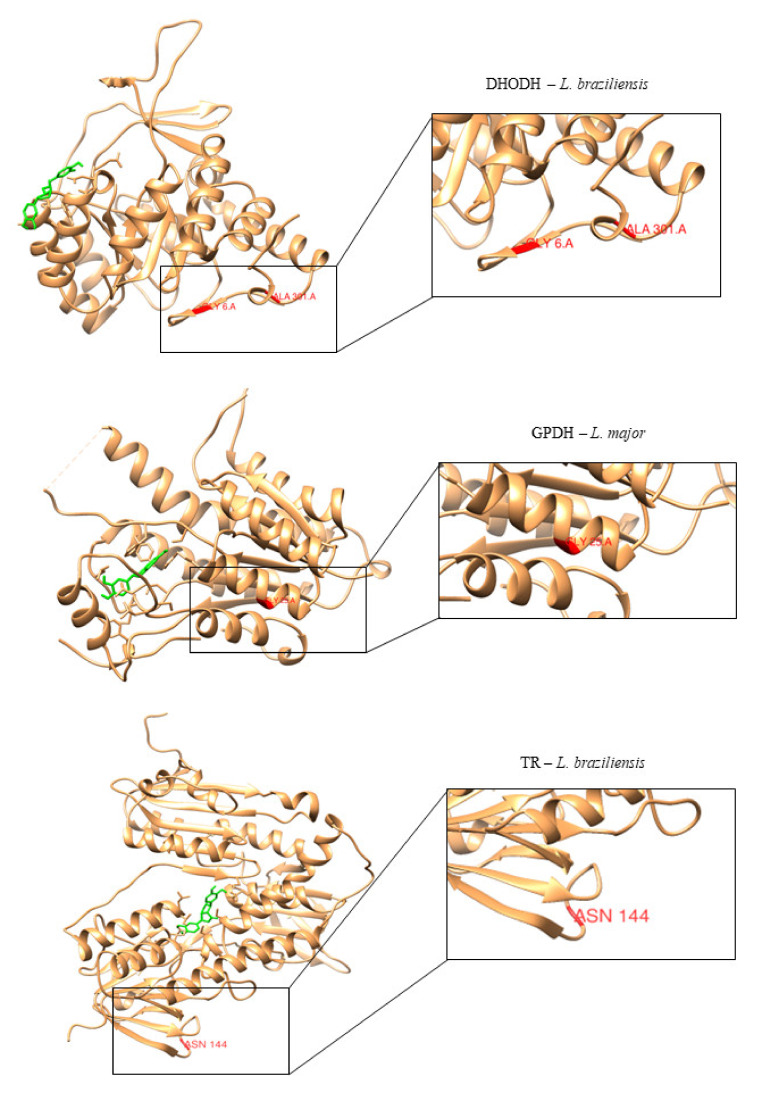
Three-dimensional structure of *L. major* and *L. braziliensis* enzymes. Amino acids that are likely to be affected by SNPs are highlighted in red. Compounds located in the active site of their respective enzymes are highlighted in green.

After identifying the primary SNPs that can cause drug resistance, we located the ancestral amino acids in the enzyme structure that are likely to be replaced by SNPs and determined whether these changes were near or in the active site of the target protein. We found that none of these amino acids were located near the active site of the studied proteins, which reduces the likelihood of drug resistance (Figure 8).

### 2.7. Molecular Dynamics Simulations

We selected some lignans that were considered to have potential multitarget leishmanicidal activity against the studied species, with excellent ADMET properties, and examined them using molecular dynamics (MD) simulations. MD assesses the flexibility of enzymes and the stability of interactions, depending on the conditions, such as solvent used, ion concentration, pressure, and temperature. Therefore, the interactions between lignans and the crystallographic ligands were used to study the flexibility and conformational changes that affect the complexes during the MD simulation. RMSD was calculated for the Cα atoms in each complexed enzyme and the structures of each ligand, separately.

**Figure 9:**
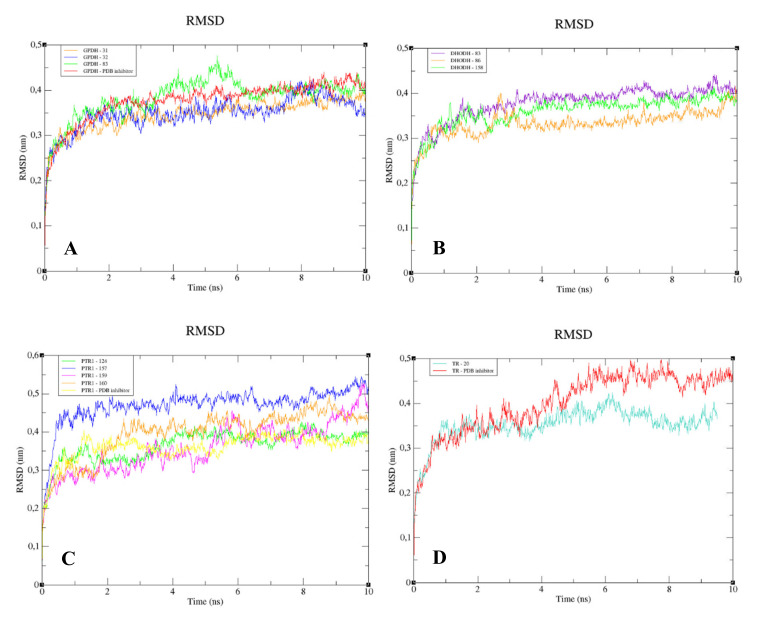

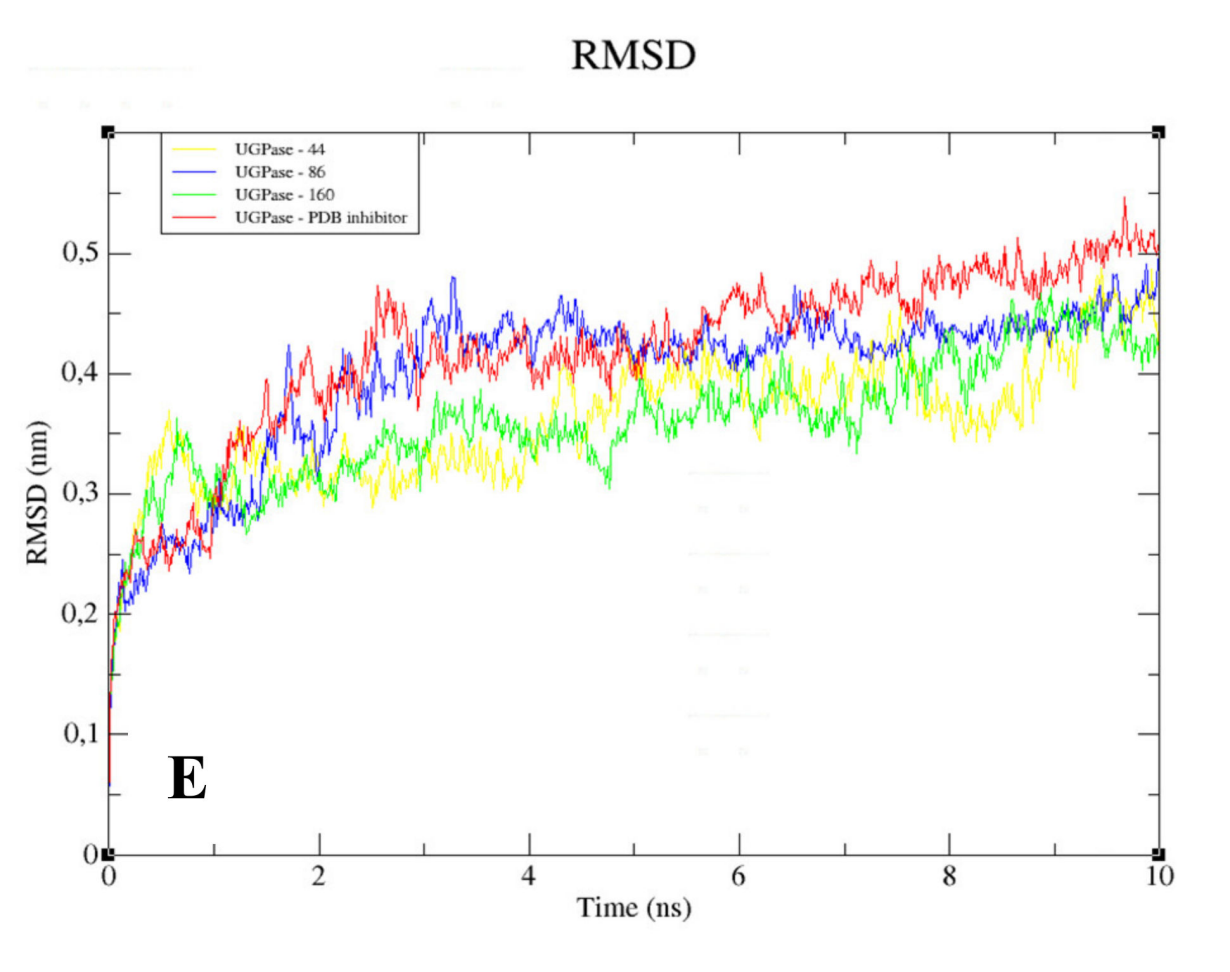
RMSD values for the Cα atoms of enzymes complexed to lignans and the Protein Data Bank (PDB) ligand. (**A**) GPDH. (**B**) DHODH. (**C**) PTR1. (**D**) TR. (**E**) UGPase.

The RMSD analysis of the complexed GPDH enzyme showed conformations ranging from 0.2 to 0.4 nm in size during 10 ns, with high stability (Figure 9A), except for GPDH complexed with compound 83, which showed a peak of instability at 5 ns but was quickly stabilized. The same pattern was observed for DHODH (Figure 9B). PTR1 also showed stability during 10 ns, except for the protein complexed with compound **159** (Figure 9C). The PTR1-157 complex was considered to be the most stable. TR complexed with lignan 20 showed greater stability than the complex containing the crystallographic ligand (Figure 9D). All complexes with the UGPase protein showed stability after 2 ns (Figure 9E). However, it was not possible to perform MD calculations for the crystallographic ligand of DHODH, due to problems with the parameterization of the ligand.

**Figure 10:**
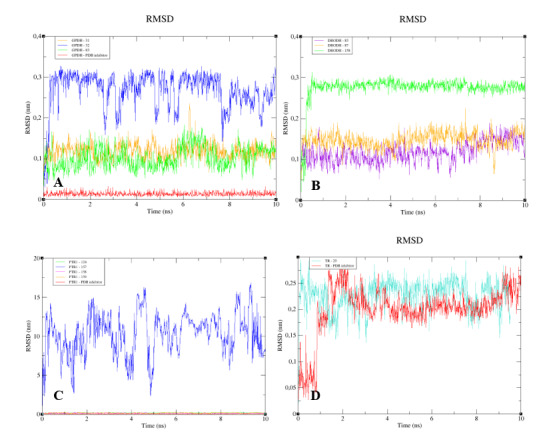

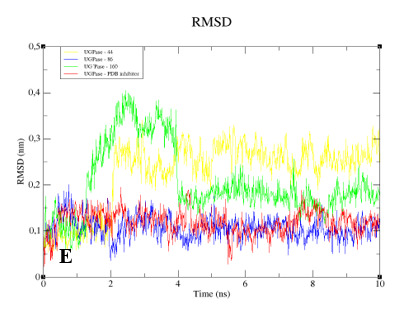
The RMSD values of the Cα atoms of the lignans and the PDB ligand. (**A**) GPDH. (**B**) DHODH. (**C**) PTR1. (**D**) TR. (**E**) UGPase.

When we analyzed the flexibility of the ligands, we found that compounds **31**, **83**, and the PDB ligand complexed with GPDH enzyme were more stable (Figure 10A). The same pattern was also found for complexes with DHODH (Figure 10B). For the PTR1 enzyme, lignan **157** showed high instability, unlike the other molecules, which remained stable (Figure 10C). Small peaks of instability were observed for TR compounds (Figure 10D). Compounds 86 and the PDB ligand showed stability during 10 ns for the UGPase protein (Figure 10E). In addition, lignan **44** achieved stability from 2 ns and lignan **160** achieved stability from 4 ns for UGPase.

**Figure 11:**
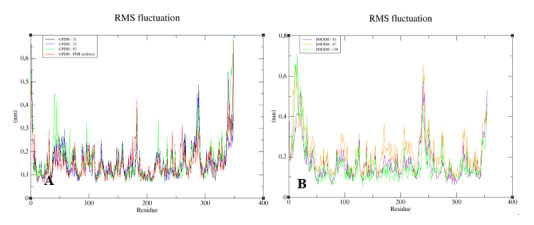

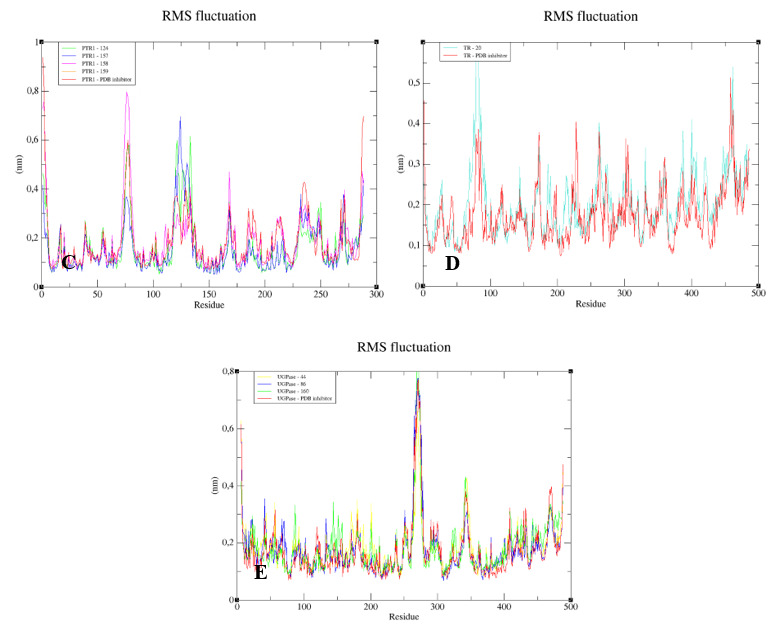
Root-mean-square fluctuation (RMSF) values for the Cα atoms of enzymes complexed with lignans and the PDB ligand. (**A**) GPDH. (**B**) DHODH. (**C**) PTR1. (**D**) TR. (**E**) UGPase.

To understand the flexibility of the residues and the amino acids that contribute to the conformational changes in the enzymes, the root-mean-square fluctuation (RMSF) values for each amino acid in each enzyme were calculated. High RMSF values suggest increased flexibility, whereas low RMSF values reflect decreased flexibility. Given that amino acids with fluctuations above 0.3 nm contribute to the flexibility of the protein structure, we found that residues at positions 1, 182, 285–290, 338–340, and 345–349 contribute to conformational changes in GPDH, with only one residue from the active site complexed to compound **83**, favoring the alteration (Figure 11A). Among the more than 300 amino acids present in DHODH, only amino acids 1, 10–25, 241–243, 250, 347, 348, and 351–354 contribute to conformational changes (Figure 11B). In PRT1, we observed that residues 1–4, 76–79, 120–123, 125–130, 132–134, 168, and 231 favor structural changes (Figure 11C). In TR, amino acids 74–88, 91, 173, 186, 351, 360, 403, 447, 448, 450, 452, 453, 456, and 458–463 favor structural changes (Figure 11D). In UGPase, the amino acids 6, 7, 43, 53, 171, 180, 201, 265, 267–274, 342, 343–346, 348, 467, 468, 487, and 488 (Figure 11E) favor structural changes. We found that none of the amino acids that affect structural conformations identified in DHODH, PTR1, TR, and UGPase are components of the active site.

### 2.8. Activity Test against L. major and L. braziliensis Axenic Promastigotes

We selected four lignans that achieved excellent results during the virtual screening process and isolated them. The compounds were evaluated for their potential to inhibit the growth of promastigotes forms of *L. major* and *L. braziliensis*. Only compound **156** was not tested in *L. major* because it did not obtain good results in silico. The results showed that lignan (**159**), epipinoresinol-4-O-β-D-glucopyranoside, displayed antileishmanial activity against *L. major* promastigotes, with an inhibitory concentration (IC_50_) of 36.51 µM (Table 12). When investigated *against L. braziliensis* promastigotes, compounds (**156**) secoisolariciresinol, (**158**) pinoresinol-4-O-β-D-glucopyranoside, (**159**) epipinoresinol-4-O-β-D-glucopyranoside, and (**160**) pinoresinol-4-O-β-D-apiofuranosyl-(1→2)-β-D-glucopyranoside inhibited growth with IC_50_ values of 9.28, 36.35, 5.39, and 13.77 µM, respectively (Table 12). The results showed that compounds **156**, **158**, and **159**, showed excellent potential as growth inhibitors during the promastigote stage. In addition, we observed that compounds (**158**), pinoresinol-4-O-β-D-glucopyranoside and (**159**), epipinoresinol-4-O-β-D-glucopyranoside, which are epimers, showed significantly different biological activities, with compound (**159**) epipinoresinol-4-O-β-D-glucopyranoside having the greatest potential against *L. braziliensis*. This result confirms the docking study consensus and combined analysis, which revealed greater potential activity for molecule **159** than for molecule **158**.

## 3. Discussion

The set of 160 Lignans and Neolignans from different subclasses (14 Furans, 10 Furofurans, 14 Dibenzylbutyrolactols, 22 Dibenzylbutanes, 21 Dibenzocycloocyadienes, 17 Aryltetralins, 3 Arylnaphthalenes, 8 Neolignans alkyl aryl ethers, 16 Neolignans benzofurans, and 9 Neolignans benzodiones) were used in this study. Lignans and Neolignans comprise a class of secondary metabolites, with diverse chemical structures, that are found in more than 70 families of plants and exhibit several significant and potent biological activities, including antioxidant [21,22], anti-inflammatory, hepatoprotective, anticancer [22,23], antimicrobial [22], trypanocidal [24,25], neuroprotective [26], and larvicidal [27] activities.

Initially, the set of 160 Lignans and Neolignans were used by various predictive parameters, which were investigated using ADMET profiles. Thus, compounds with better ADMET profiles were subjected to further virtual screening methodologies.

The RF model was able to select 11 compounds with active potentials ranging from 50% to 57%, for *L. major*, and 21 potentially active compounds for *L. braziliensis*, with probabilities ranging between 50% and 75%. We observed the probabilities for biological activity were higher for *L. braziliensis;* therefore, we suggest that lignans may offer greater therapeutic potential against this species.

Virtual ligand-based screening is a method that is capable of evaluating and/or selecting compounds with desired properties, using chemical structures associated with known biological activity data to develop models, such as QSAR analyses [29]. QSAR models contribute to planning and drug development by reducing the costs of new molecule development and reducing the number of animals necessary for experimental tests.

Docking is a virtual screening method, based on structure that can identify selective compounds and predict the mechanisms of action. We performed consensus docking for the molecules that were identified as potentially active by the RF models. The consensus docking method allows the elimination of potential false positives and ensures the increased reliability of the procedure. The results showed that all of the lignans identified as potentially active by the RF models achieved excellent results during the consensus docking analyses for different enzymes.

When analyzing the results of consensus docking, we observed that the same compounds were ranked among the first five positions in one docking program, whereas it was listed among the bottom positions in another program. In addition, when standardizing the docking results, we identified a compound that failed in three programs; however, when all the binding energies were averaged, that compound increased in rank. These results were due to a single program assigning this molecule much higher energy values than the other programs. Therefore, we used two criteria for the elimination of compounds during the consensus docking analysis: i) Compounds that failed in at least three docking programs; and ii) compounds that obtained average consensus docking energy values below that of the crystallographic ligand.

Understanding the performance of each docking program is necessary to verify the quality and reliability of the virtual screening process, regardless of whether a consensus approach is applied. Therefore, the use of a consensus strategy and the performance evaluation of the docking programs were essential for our structure-based virtual screening analysis.

A study performed by Chang et al. [30] compared the virtual screening results obtained for HIV protease inhibitors, using AutoDock 4 and Vina. The authors concluded that both AutoDock 4 and Vina were able to select active compounds (AUC = 0.69 and 0.68, respectively; *p* < 0.001) and that Vina was more scalable for the treatment of difficult coupling problems (i.e., larger and more flexible compounds) than AD4. Another study, recently performed by Ren et al. [31], investigated the performance of several docking programs, to validate a scoring function strategy, and observed that among the evaluated programs, Plants and Vina obtained success rates of 53.6% and 48.5%, respectively. In contrast, the Gold program achieved success rates of up to 81% (within 2.0 Å of the experimental binding mode), according to Verdonk et al. [32] and was more than 60% successful at predicting the correct poses for 84 evaluated protein-ligand complexes, in a study by Chaput and Mouawad [33].

The combined analysis, based on both ligand and structure, allowed the selection of several potentially active and multitarget compounds against three or more enzymes, except lignan **76**, which obtained good results against only two enzymes. We noticed that the combined probability values were better for the species *L. braziliensis*, for which most of the lignans showed Prob_Comb_ values between 60% and 80% of activity. These results were confirmed by in vitro assays, using *L. braziliensis* promastigotes, as all tested molecules inhibited parasite growth. In *L. major*, of the four compounds tested, only one achieved antileishmanial activity. We, therefore, suggest that a probability of activity greater than 60% is necessary to inhibit growth.

An interesting study by Stevanovic et al. [34] found success in their study using homology detection methods and in silico screening to search for potential inhibitors of a new target, type 2 NADH dehydrogenase of *Leishmania infantum*. According to the researchers, the selected compounds that exhibited favorable properties in the computational screening experiments were tested and the leishmanicidal activity was determined in amastigotes and wild-type axenic promastigotes of *L. infantum*. The results showed that the identified compound, a substituted 6-methoxy-quinalidine, showed promising activity under the two cellular forms.

Although the isolated compounds have been tested only in amastigote forms, several studies indicate that the enzymes addressed in the study are expressed in amastigote and promastigote forms and there is proven biological activity for these enzymes in both forms. According to Choe et al. [26], the enzyme GPDH is more expressed especially in the amastigote form, where fatty acids, instead of carbohydrates, are predominantly used as an energy source. According to Steiner et al. [35], glucose UDP is essential for all organisms and in *Leishmania*, several gluconjugates are expressed during the parasite’s life cycle, allowing survival and proliferation in the vector and in the mammalian host. Experimental data for the in vitro inhibition of *Lm*DHODH indicate that natural products can actually inhibit *Lm*DHODH against promastigotes and amastigotes [36]. The same study identified several secondary metabolites that were able to inhibit *Lm*DHODH in vitro at concentrations of IC_50_ 27 μM, 30 μM and 31 μM. Compounds based on a structure-guided approach designed to have anti-leishmanial activity through the anti-folate mechanism, targeting *Lm*PTR1 in vitro was promising for promastigote and amastigote forms with IC_50_ values of 4.2 μM and 3.3 μM, respectively [37]. Da Sila et al. [38] reported inhibitory activity of the compound ResAn2 for the target TR of *L. braziliensis* against promastigotes and amastigotes with IC_50_ values of 10.27 μM and 17.54 μM.

We also observed that three of the four isolated lignans, which showed inhibitory activity for *L. braziliensis*, belong to the furofurans class. In addition, these lignans have glucose units attached to their structures, which can enhance their therapeutic action. According to Xu et al. [39], furofurans are lignans originally formed by the enantioselective dimerization of two units of coniferyl alcohol derived from the biosynthetic shiquimate pathway. Furofuran lignans are known to have a diversity of structures due to bonding patterns, different substituents, and different configurations [39]. This diversity contributes to a variety of biological activities, including anti-cancer [40], antioxidants, anti-inflammatory, cytotoxic, antimicrobial [39], and antiestrogenic [41]. Therefore, this research brings new information about antileishmanial activity for this class of lignans.

## 4. Materials and Methods

**Figure 12:**
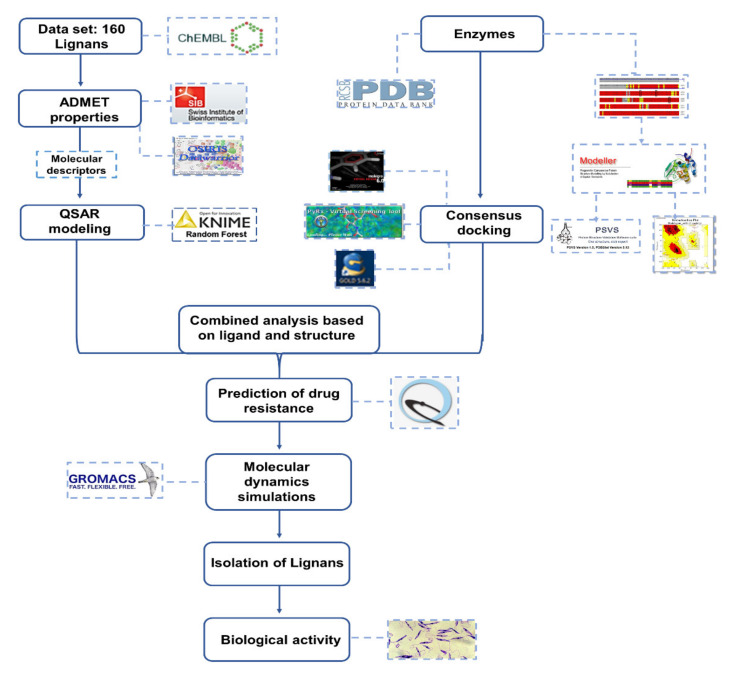
Scheme of all procedures used in this study.

Figure 12 shows a schematic depicting all of the methodologies used in this study.

### 4.1. Predicting ADMET Properties

ADME parameters were calculated using the SwissADME open-access web tool (Swiss Institute of Bioinformatics, Switzerland, http://www.swissadme.ch), whereas the toxicity prediction was performed in the OSIRIS Property Explorer (Idorsia Pharmaceuticals Ltd., Allschwil, Switzerland, https://www.organic-chemistry.org/prog/peo/) [42]. For absorption, factors including membrane permeability and intestinal absorption were considered. We also investigated compounds that did not exceed more than two violations of the Lipinski rule and for which the logP consensus value was not greater than 4.15. The distribution was assessed by factors that included the blood–brain barrier (logBB) and the permeability of the central nervous system (CNS). Metabolism was predicted based on the substrate models of cytochrome P (CYP). Compounds that were substrates or inhibited more than two enzymes (CYP1A2, CYP2C19, CYP2C9, CYP2D6, CYP3A4) were eliminated. The toxicity of the drug was predicted based on the following parameters: Mutagenicity, tumorigenicity, reproductive effects, and irritability.

### 4.2. Data Collection and Curation

Chemical compounds with known activity (pIC_50_) against *L. major* (CHEMBL612879) and *L. braziliensis* (CHEMBL612878) (EMBL-EBI, Wellcome Genome Campus, Cambridgeshire, England) were obtained from the ChEMBL database (https://www.ebi.ac.uk/chembl/) [43] for the construction of predictive models. In addition, we obtained 160 lignans e neolignans from ChEMBL to use during virtual screening for the identification of compounds with leishmanicidal potential. All compounds were selected for chemical and biological data, according to the workflows established by Fourches et al. [44,45]. A duplicate search was performed using the HiT QSAR software (Hierarchical QSAR technology, Ukraine). The 3D structures were generated by ChemaxonStandardizer v.18.17.0, (ChemAxon, Boston, USA, www.chemaxon.org).

### 4.3. QSAR Modeling

Knime 3.6.2 software (Knime 3.6.2, Copyright Miner, from Konstanz Information, Zurich, Switzerland, www.knime.org) was used to perform QSAR modeling. Given the success of our previous studies [46,47], we opted to perform a QSAR 3D analysis. For this, all compounds with a solved chemical structure were saved in SDF format and imported into Dragon 7.0 software (Kode Chemoinformatics srl, Pisa, Italy) [48], to generate descriptors. The RF algorithm was used to build prediction models. The applicability domain was estimated, according to procedures described [49]. External cross-validation was performed, to estimate the predictive power of the models developed. In addition, the performance of external models was assessed by ROC analysis. The models were also analyzed using MCC, to evaluate the model globally, based on the results obtained from the confusion matrix.

### 4.4. Alignment of Protein Sequences

The 3D sequences and structures of GPDH, DHODH, PTR1, TR, and UGPase in *L. major* and *L. braziliensis* were obtained from the GenBank database (National Center for Biotechnology Information, Bethesda MD, USA, https://www.ncbi.nlm.nih.gov/) [50].

Then, a global alignment was performed, using the web tool Clustal Omega (EMBL-EBI, Cambridgeshire, UK, (https://www.ebi.ac.uk/Tools/msa/clustalo/), which aligns all protein sequences entered by a user. The alignment facilitated the investigation of the active site and the determination of the similarity and shared identity among the enzymes between the two species of Leishmania.

### 4.5. Homology Modeling

The sequences of the enzymes and species selected in the study were obtained from the GenBank database (National Center for Biotechnology Information, Bethesda MD, USA, https://www.ncbi.nlm.nih.gov/) [50], and the template structures were obtained from the Protein Data Bank (PDB, https://www.rcsb.org/pdb/home/home.do) [51]. Four enzymes were selected for the construction of homology models: GPDH, PTR1, TR, and UGPase. The template enzymes were: GPDH from *L. mexicana* (PDB ID: 1M66), PTR1 from *L. major* (PDB ID: 5L42), TR from L*. infantum* (PDB ID: 5EKB), and UGPAse from *L. major* (PDB ID: 5NZM). The enzyme models were constructed using the homology molecular modeling method in MODELLER 9.20 software (copyright © 2020-2020 Andrej Sali, maintained by Ben Webb at the Departments of Biopharmaceutical Sciences and Pharmaceutical Chemistry, and California Institute for Quantitative Biomedical Research, Mission Bay Byers Hall, University of California San Francisco, San Francisco, USA) [52]. Five models were generated, and the lowest energy model was chosen. The stereochemical qualities of the model were assessed by the PSVS webserver (Protein Structure Validation Software suite) (http://psvs-1_5-dev.nesg.org/), using PROCHECK [53]. PROCHECK generates a Ramachandran graph [54], which determines the permitted and disallowed regions of the main chain of amino acids. The structural quality was evaluated using the VERIFY 3D software (saves @2020 - DOE-MBI Services, http://services.mbi.ucla.edu/SAVES/), and the compatibility between the protein sequence and its 3D structure, based on the chemical environment, was analyzed using WHAT IF (http://swift.cmbi.ru.nl/servers/html/index.html).

### 4.6. Consensus Docking

The consensus docking analysis was performed using four different packages: Molegro Virtual Docker (MVD) (Molexus IVS Rørth Ellevej 3, Odder, Denmark) [55], GOLD 5.6.2 (The Cambridge Crystallographic Data Centre, Cmabridge, USA) [32], AutoDock Vina (Vina) (Molecular Graphics Lab at The Scripps Research Institute) [56,57], and AutoDock 4.2.6. (AD4) (The Scripps Research Institute, La Jolla, USA), with standard parameters [58]. Five scoring functions were selected for the consensus analysis, including Moldock score (Molexus IVS Rørth Ellevej 3, Odder, Denmark), Goldscore (The Cambridge Crystallographic Data Centre, Cmabridge, USA), ChemPLP (The Cambridge Crystallographic Data Centre, Cmabridge, USA), and the Binding affinity scores in Vina (Molecular Graphics Lab at The Scripps Research Institute) and (The Scripps Research Institute, La Jolla, USA). The enzymes constructed by homology and selected from PDB were used for docking analysis. Information on the enzymes obtained from PDB and their respective inhibitors can be found in Table 13. Initially, all water molecules were removed from the crystalline structure. RMSD ≤ 2.0 Å was used as a criterion for docking success. The consensus strategy consisted of selecting compounds with higher binding affinity prediction values than those for the crystallographic ligands, based on at least three different scoring functions. Then, the values were standardized and averaged. This approach increases the reliability of the fit and increases the number of true positive compounds.

### 4.7. Prediction of Drug Resistance

Genetic variations in the target enzymes were analyzed by searching the TritrypDB database (VEuPathDB, USA, http://tritrypdb.org/tritrypdb/) to identify SNPs in *L. major* and *L. braziliensis*.

After the identification of SNPs, their presence in the region of the active site was investigated, and the most prevalent mutations in the active site or near them were examined, to verify whether these mutations resulted in structural changes or interfered with compound interactions. The mutations were designed using the UCSF Chimera program (Visualization and Informatics – RBVI, San Francisco, USA) [59]. Then, MVD docking was performed, to assess the binding affinity with lignans in the presence of mutations.

### 4.8. Molecular Dynamics Simulations

MD simulations were performed to estimate the flexibility of interactions between proteins and ligands, using GROMACS 5.0 software (European Union Horizon 2020 Programme, Sweden) [60,61]. The protein and ligand topologies were also prepared using the GROMOS96 54a7 force field. The MD simulation was performed using the SPC water model of point load, extended in a cubic box [62]. The system was neutralized by the addition of ions (Cl^−^ and Na^+^) and minimized, to remove bad contacts between complex molecules and the solvent. The system was also balanced at 300K, using the 100 ps V-rescale algorithm, represented by NVT (constant number of particles, volume, and temperature), followed by equilibrium at 1 atm of pressure, using the Parrinello-Rahman algorithm as the NPT (constant pressure particles and temperature), up to 100 ps. DM simulations were performed in 5,000,000 steps, at 10 ns. To determine the flexibility of the structure and whether the complex is stable close to the experimental structure, RMSD values of all Cα atoms were calculated relative to the starting structures. RMSF values were also analyzed, to understand the roles played by residues near the receptor binding site. The RMSD and RMSF graphs were generated in Grace software (Grace Development Team, http://plasma-gate.weizmann.ac.il/Grace/).

### 4.9. Isolation and Identification of Lignans

The compounds secoisolariciresinol [63], pinoresinol-4-*O*-β-d-glucopyranoside [64], pinoresinol-4-*O*-β-d-apiofuranosyl-(1→2)-β-d-glucopyranoside [65], epipinoresinol-4-*O*-β-d-glucopyranoside [66] and were isolated from the fractionation of the crude ethanolic extract of thespecies *Justicia aequilabris* (Nees) Lindau [67] collected in the city of Puxinanã-PB, Brazil, registered in SisGen under the number: A35A42B. The compounds were identified by nuclear magnetic resonance and high-resolution mass spectrometry (HRMS).

Furofuran lignans have their configuration well established in literature, according to Shao et al. [66], the 7, 9′:7′, 9-diepoxi moiety of furofuran lignans of natural origin occurs in the cis-fused configuration. The experiments carried out have demonstrated that chemical deviations of the ΔδH-9 and ΔδH-9′ are resulted of relative configurations of the C-7/C-8 and C-7′/C-8′. Therefore, the authors present the values for chemical displacement differences H2-9 and H2-9′ in different solvents. Thus, compound 158 has the value of ΔδH-9; H-9′ both = 0.40, classifying this molecule as 7-H/8-H trans, 7′-H/8′-H trans. Substance 159 showed ΔδH-9 = 0.34 and ΔδH-9′ = 0.65, determining 7-H/8-H cis and 7′-H/8′-H trans positions. Structure 160 showed Δδ H-9; H-9′ = 0.40, indicating 7-H/8-H and 7′-H/8′-H as trans configuration. All the compounds were analyzed on DMSO-d6. The absolute configuration of compound 156 was deduced by utilizing the experimental and calculated electronic circular dichroism (ECD) data, assigned to the molecule the 8R and 8′R configuration.

### 4.10. Activity Tests against L. major and L. braziliensis Axenic Promastigotes

*L. major* (MHOM/IL/80/Friedlin) and *L. braziliensis* (MHOM/BR/1975/M2903) species were maintained in vitro, as promastigotes, at 26 °C in supplemented Schneider insect medium (20% SFB, 100 U/mL penicillin and 100 µg/mL streptomycin, pH 7), as described by Rodrigues et al. [68]. The growth inhibition assay for the parasites was performed using promastigote forms in the logarithmic phase, which were grown in 96-well plates containing supplemented Schneider insect medium and 1 × 106 parasites/mL, and was performed in triplicate, using different concentrations of lignans (1.56–12.5 µM) and reference meglumine antimoniate drugs (200–40,000 µM) and amphotericin B (0.031–2 µM). The negative control contained neither the reference nor the tested compounds. The culture plates were maintained in a biological oxygen demand incubator (Eletrolab EL202, São Paulo, Brazil), at 26 °C, for axenic promastigotes. After 2 days under these conditions, 10 µL of 3-(4,5-Dimethylthiazol-2-yl)-2,5-Diphenyltetrazolium Bromide (MTT, 5 mg/mL) was added to each well, and the cell culture plates were incubated for 4 h before adding 50 µL of 10% sodium dodecyl sulfate (SDS) solution, to solubilize the formazan crystals. The optical density of the culture was measured in a microplate spectrophotometer reader, at 540 nm (Biosystems ELx800 model, Curitiba, Brazil).

## 5. Conclusions

Leishmaniasis is endemic in more than 90 countries, affecting low-income populations. Leishmaniasis is estimated to affect 2 million people annually, worldwide, and more than 20,000 deaths per year are due to complications from the disease. *L. major* and *L. braziliensis* are responsible for CL, which represents more than 90% of cases in several countries. Because current treatments can result in drug-resistance and are often associated with side effects, due to high toxicity index values, lignans have been investigated as a treatment alternative, particularly because many lignans have great ADMET profiles.

We used simple programs and rules to calculate the absorption, bioavailability, pharmacokinetics, and to select lignans with good properties. We were able to screen 33 promising lignans from a set of 160 compounds, which were subjected to several computational and experimental approaches to investigate their leishmanicidal potentials.

The generated predictive models obtained excellent performance results, with an accuracy greater than 81%, and selected 11 lignans with active potential probabilities ranging between 50% and 57%, for *L. major.* For *L. braziliensis*, an accuracy greater than 79% was achieved, and the model selected 21 lignans, with activity probabilities between 50% and 75%.

To investigate the mechanism of action for lignans and to evaluate their selectivity for five enzymes in Leishmania, a consensus docking analysis was performed, to guarantee the reliability of the RF model and to reduce the number of false positives. Eleven lignans were found to be potentially active against *Lm*GPDH, 10 against *Lm*DHODH, 9 against *Lm*PTR1, 9 against *Lm*TR, and 11 against *Lm*UGPase. Among the 21 lignans analyzed in *L. braziliensis*, 20 were potentially active against *Lb*GPDH, 21 against *Lb*DHODH, 19 against *Lb*PTR1, 16 against *Lb*TR, and 15 against *Lb*UGPase.

A combined analysis, based on both ligand and structure, was able to identify potentially active molecules, using both RF and multitarget models, resulting in the identification of 23 potentially active, multitarget molecules against *L. major* and/or *L. braziliensis*, with 10 compounds common to both species.

Due to concerns regarding the development of drug resistance during the treatment of Leishmaniasis, the present study investigated the presence of SNPs, which may contribute to the development of drug resistance. Among the 14 SNPs identified, only four presented polymorphic alleles with relevant frequencies, between 40% and 50%. We also found that none of the amino acids affected by the SNPs were located near the active sites of studied proteins, which reduces the likelihood of developing drug resistance.

MD simulations revealed that most of the studied enzymes are stable under various conditions, including various solvents, ions, temperatures, and pressure, with only small variations observed for some complexed compounds. Therefore, the binding affinity between proteins and ligands is unlikely to be affected by environmental changes. In addition, none of the amino acids responsible for the enzymatic conformational changes were in the active site, except for those in the DHODH-83 complex, which allows the active site to remain stable.

Four lignans with excellent ADMET profiles, which are considered to be potentially active and multitarget inhibitors for the studied enzymes, were isolated from Justicia aequilabris (Nees) Lindau [65] and subjected to in vitro tests. Lignans were collected in the city of Puxinanã-PB, Brazil. The results showed that only lignan (**159**) epipinoresinol-4-O-β-d-glucopyranoside achieved antileishmanial activity against promastigotes forms of L. major, with an IC_50_ value of 36.51 µM. In L. braziliensis, compounds (**156**) secoisolariciresinol, (**158**) pinoresinol-4-O-β-d-glucopyranoside, (**159**) epipinoresinol-4-O-β-d-glucopyranoside, and (**160**) pinoresinol-4-O-β-d-apiofuranosyl-(1→2)-β-d-glucopyranoside inhibited growth, with IC_50_ values of 9.28, 36.35, 5.39, and 13.77, respectively. Compounds (**156**) secoisolariciresinol, (**159**) epipinoresinol-4-O-β-d-glucopyranoside, and (**160**) pinoresinol-4-O-β-d-apiofuranosyl-(1→2)-β-d-glucopyranoside showed excellent potential as growth inhibitors for the promastigote stage of the parasite. When compared with the values obtained from the biological activity prediction, using the RF models, we noticed that the probability of activity for L. major varied 50%–60%, whereas those values for L. braziliensis ranged 50%–75%. We suggest that it may be preferable to test compounds with probabilities of activity above 60% to obtain good results and that the computational approach can be used to guide experimental research.

## Figures and Tables

**Table 1:** Summary of parameters corresponding to the results obtained for all models.

Specie	Validation	Specificity	Sensitivity	Accuracy	PPV	NPV	MCC
*L. major*	Test	0.81	0.82	0.81	0.83	0.79	0.63
	Cross	0.80	0.83	0.82	0.83	0.80	0.63
*L. braziliensis*	Test	0.83	0.73	0.79	0.76	0.80	0.57
	Cross	0.85	1	0.91	0.83	1	0.87

**Table 2:** Lignans activity probabilities (pActivity) against *L. major*, as assessed by the RF model.

ID	IUPAC Name	pActivity
**44**	(1*R*,2*S*)-2-(2,6-Dimethoxy-4-prop-2-enylphenoxy)-1-(4-hydroxy-3,5-dimethoxyphenyl)propane-1,3-diol	0.54
**60**	(1*S*,2*S*)-1-(4-Hydroxy-3-methoxyphenyl)-2-[4-(3-hydroxypropyl)-2-methoxyphenoxy]propane-1,3-diol	0.53
**83**	4-[(2*S*,3*R*)-5-[(*E*)-3-Hydroxy-3-methoxyprop-1-enyl]-3-(hydroxymethyl)-7-methoxy-2,3-dihydro-1-benzofuran-2-yl]-2-methoxyphenol	0.55
**86**	(*E*)-3-[(2*R*,3*S*)-2-(3,4-Hihydroxyphenyl)-3-(hydroxymethyl)-2,3-dihydro-1,4-benzodioxin-6-yl]prop-2-enoic acid	0.57
**87**	4-[(2*R*,3*R*)-3-(Hydroxymethyl)-6-(3-hydroxypropyl)-2,3-dihydro-1,4-benzodioxin-2-yl]-2-methoxyphenol	0.51
**124**	Ethyl (2*S*,3*R*)-2-(3,4-dihydroxyphenyl)-5-[(*E*)-3-ethoxy-3-oxoprop-1-enyl]-7-hydroxy-2,3-dihydro-1-benzofuran-3-carboxylate	0.52
**132**	(3*R*,4*S*)-4-[(*R*)-(4-Hydroxy-3-methoxyphenyl)[(5*R*)-4-hydroxy-5-methoxycyclohexa-1,3-dien-1-yl]methyl]-3-(hydroxymethyl)oxolan-2-one	0.53
**157**	(2*S*,3*S*,4*R*,5*R*,6*S*)-2-{4-[(2*S*,3*R*,4*S*)-4-[(4-Hydroxy-3-methoxyphenyl)methyl]-3-(hydroxymethyl)oxolan-2-yl]-2-methoxyphenoxy}-6-(hydroxymethyl)oxane-3,4,5-triol	0.53
**158**	(2*R*,3*S*,4*R*,5*R*,6*S*)-2-{4-[(1*S*,3a*R*,4*S*,6a*R*)-4-(4-hydroxy-3-methoxyphenyl)-hexahydrofuro[3,4-c]furan-1-yl]-2-methoxyphenoxy}-6-(hydroxymethyl)oxane-3,4,5-triol	0.50
**159**	(2*R*,3*S*,4*R*,5*R*,6*S*)-2-{4-[(1*S*,3a*R*,4*R*,6a*R*)-4-(4-hydroxy-3-methoxyphenyl)-hexahydrofuro[3,4-c]furan-1-yl]-2-methoxyphenoxy}-6-(hydroxymethyl)oxane-3,4,5-triol	0.50
**160**	(2*S*,3*R*,4*R*,5*S*,6*R*)-6-{4-[(1*S*,3a*R*,4*S*,6a*R*)-4-(4-hydroxy-3-methoxyphenyl)-hexahydrofuro[3,4-c]furan-1-yl]-2-methoxyphenoxy}-5-{[(2*S*,3*R*,4*R*)-3,4-dihydroxy-4-(hydroxymethyl)oxolan-2-yl]oxy}-2-(hydroxymethyl)oxane-3,4-diol	0.56

**Table 3:** Lignans activity probabilities (pActivity) against *L. braziliensis*, as assessed by the RF model.

ID	IUPAC Name	pActivity
**8**	(1*S*,5*S*,6*R*,7*S*,8*R*)-7-(1,3-Benzodioxol-5-yl)-1,3,8-trihydroxy-6-methyl-5-prop-2-enylbicyclo[3.2.1]oct-3-en-2-one	0.70
**20**	4-[[(3*R*,4*R*,5*S*)-5-(4-Hydroxy-3-methoxyphenyl)-4-(hydroxymethyl)oxolan-3-yl]methyl]-2-methoxyphenol	0.60
**31**	(2*R*,3*R*,4*R*)-3,4-bis[(4-Hydroxy-3-methoxyphenyl)methyl]axonal-2-ol	0.53
**32**	(2*S*,3*R*,4*R*)-3,4-bis[(4-Hydroxy-3-methoxyphenyl)methyl]axonal-2-ol	0.54
**44**	(1*R*,2*S*)-2-(2,6-Dimethoxy-4-prop-2-enylphenoxy)-1-(4-hydroxy-3,5-dimethoxyphenyl)propane-1,3-diol	0.59
**57**	(1*R*,2*R*)-1-(4-Hydroxy-3-methoxyphenyl)-2-[4-(3-hydroxypropyl)-2-methoxyphenoxy]propane-1,3-diol	0.67
**58**	4-[3-Hydroxy-2-[1-(4-hydroxy-3-methoxyphenyl)-1-methoxybutan-2-yl]oxypropyl]-2,6-dimethoxyphenol	0.68
**59**	4-[2-[1-Hydroxy-3-(4-hydroxy-3-methoxyphenyl)propan-2-yl]oxy-1-methoxybutyl]-2,6-dimethoxyphenol	0.64
**60**	(1*S*,2*S*)-1-(4-Hydroxy-3-methoxyphenyl)-2-[4-(3-hydroxypropyl)-2-methoxyphenoxy]propane-1,3-diol	0.70
**61**	4-[(3*S*,3a*R*,6*S*,6a*R*)-6-(3,4,5-Trimethoxyphenyl)-1,3,3a,4,6,6a-hexahydrofuro[3,4-c]furan-3-yl]-2,6-dimethoxyphenol	0.64
**76**	(9*R*,10*R*,11*R*)-11-Hydroxy-3,4,5,19-tetramethoxy-9,10-dimethyl-15,17-dioxatetracyclononadeca-1(19),2(7),3,5,12,14(18)-hexene-6-carboxylic acid	0.52
**83**	4-[(2*S*,3*R*)-5-[(*E*)-3-Hydroxy-3-methoxyprop-1-enyl]-3-(hydroxymethyl)-7-methoxy-2,3-dihydro-1-benzofuran-2-yl]-2-methoxyphenol	0.62
**87**	4-[(2*R*,3*R*)-3-(hydroxymethyl)-6-(3-hydroxypropyl)-2,3-dihydro-1,4-benzodioxin-2-yl]-2-methoxyphenol	0.5
**91**	4-[[(2*R*,3*R*,4*R*)-4-[(*S*)-Hydroxy-(4-hydroxy-3-methoxyphenyl)methyl]-2-methoxyoxolan-3-yl]methyl]-2-methoxyphenol	0.65
**121**	(2*R*,3*R*,4*R*)-2-(4-Hydroxy-3-methoxyphenyl)-4-[(4-hydroxy-3-methoxyphenyl)methyl]-3-(hydroxymethyl)oxolan-3-ol	0.61
**124**	Ethyl (2*S*,3*R*)-2-(3,4-dihydroxyphenyl)-5-[(*E*)-3-ethoxy-3-oxoprop-1-enyl]-7-hydroxy-2,3-dihydro-1-benzofuran-3-carboxylate	0.59
**156**	(2*R*,3*R*)-2,3-bis[(4-Hydroxy-3-methoxyphenyl)methyl]butane-1,4-diol	0.57
**157**	(2*S*,3*S*,4*R*,5*R*,6*S*)-2-{4-[(2*S*,3*R*,4*S*)-4-[(4-Hydroxy-3-methoxyphenyl)methyl]-3-(hydroxymethyl)oxolan-2-yl]-2-methoxyphenoxy}-6-(hydroxymethyl)oxane-3,4,5-triol	0.75
**158**	(2*R*,3*S*,4*R*,5*R*,6*S*)-2-{4-[(1*S*,3a*R*,4*S*,6a*R*)-4-(4-hydroxy-3-methoxyphenyl)-hexahydrofuro[3,4-c]furan-1-yl]-2-methoxyphenoxy}-6-(hydroxymethyl)oxane-3,4,5-triol	0.66
**159**	(2*R*,3*S*,4*R*,5*R*,6*S*)-2-{4-[(1*S*,3a*R*,4*R*,6a*R*)-4-(4-hydroxy-3-methoxyphenyl)-hexahydrofuro[3,4-c]furan-1-yl]-2-methoxyphenoxy}-6-(hydroxymethyl)oxane-3,4,5-triol	0.66
**160**	(2*S*,3*R*,4*R*,5*S*,6*R*)-6-{4-[(1*S*,3a*R*,4*S*,6a*R*)-4-(4-hydroxy-3-methoxyphenyl)-hexahydrofuro[3,4-c]furan-1-yl]-2-methoxyphenoxy}-5-{[(2*S*,3*R*,4*R*)-3,4-dihydroxy-4-(hydroxymethyl)oxolan-2-yl]oxy}-2-(hydroxymethyl)oxane-3,4-diol	0.72

**Table 4:** Percentage of amino acids present in the permitted and favored regions of the Ramachandran chart for each model.

Enzyme	Species	Ramachandran Percentage
GPDH	*L. braziliensis*	100%
	*L. major*	100%
TR	*L. braziliensis*	98%
	*L. major*	98%
PTR1	*L. braziliensis*	100%
UGPase	*L. braziliensis*	100%

**Table 5:** Percentage of the degree of compatibility between the 3D structure and the 1D amino acid sequence, based on Verify 3D generated models.

Enzyme	Species	Verify 3D Percentage
GPDH	*L. braziliensis*	91.83%
	*L. major*	85.56%
TR	*L. braziliensis*	90.22%
	*L. major*	93.69%
PTR1	*L. braziliensis*	80.56%
UGPase	*L. braziliensis*	93.47%

**Table 6:** Average scores for each residue, obtained from the WHAT IF server, for each model.

Enzyme	Species	Average WHAT IF Score
GPDH	*L. braziliensis*	−1.622
	*L. major*	−1.578
TR	*L. braziliensis*	−0.919
	*L. major*	−0.894
PTR1	*L. braziliensis*	−0.952
UGPase	*L. braziliensis*	−0.609

**Table 7:** Combined probabilities between prediction models and molecular docking analysis for potential activity against *L. major*.

ID	P_Activity_	Prob_Comb_				
		GPDH	DHODH	PTR1	TR	UGPase
**44**	0.54	0.64	0.60	0.65	0.68	0.68
**60**	0.53	0.66	0.58	0.63	0.66	0.66
**83**	0.55	0.67	0.59	0.67	0.68	0.68
**86**	0.57	0.68	0.62	0.68	0.69	0.69
**87**	0.51	0.65	0.58	-	0.65	0.65
**124**	0.52	0.56	0.57	0.66	0.69	0.69
**132**	0.53	0.66	0.57	-	0.66	0.66
**157**	0.53	0.68	0.63	0.65	-	0.60
**158**	0.50	0.66	0.59	0.66	-	0.58
**159**	0.50	0.64	0.63	0.69	0.60	0.66
**160**	0.56	0.71	0.65	0.71	0.63	0.63

**Table 8:** Combined probabilities between prediction models and molecular docking analysis for potential activity against *L. braziliensis*.

ID	P_Activity_	Prob_Comb_				
		GPDH	DHODH	PTR1	TR	UGPase
**8**	0.70	0.75	0.66	0.73	-	-
**20**	0.60	0.69	0.62	0.68	0.66	-
**31**	0.53	0.65	0.57	0.65	0.62	0.62
**32**	0.54	0.65	0.57	0.65	0.61	0.62
**44**	0.59	0.62	0.61	0.71	0.65	0.66
**57**	0.67	0.72	0.65	0.75	0.69	0.71
**58**	0.68	0.70	0.65	0.71	0.69	0.72
**59**	0.64	0.67	0.63	0.69	-	0.70
**60**	0.70	0.74	0.67	0.75	0.71	0.74
**61**	0.64	0.71	0.66	0.65	0.68	-
**76**	0.52	-	0.55	-	-	-
**83**	0.62	0.72	0.67	0.72	0.70	0.69
**87**	0.5	0.63	0.55	0.60	0.60	0.59
**91**	0.65	0.74	0.65	0.73	0.69	0.69
**121**	0.61	0.71	0.62	0.69	-	-
**124**	0.59	0.67	0.62	0.70	0.69	-
**156**	0.57	0.64	0.61	0.66	0.62	0.66
**157**	0.75	0.79	0.78	0.84	0.77	0.78
**158**	0.66	0.77	0.68	0.73	0.72	0.72
**159**	0.66	0.77	0.68	0.75	0.77	0.77
**160**	0.72	0.76	0.81	0.77	-	0.80

**Table 9:** Information on the crystalline structures and the root-mean-square deviation (RMSD) values for the poses obtained by redocking.

Protein-Ligand Complex			RMSD				
Enzyme	PDB ID	Inhiibidor	Moldoscore	Goldscore	Vina	AD4	ChemPLP
GPDH	1M66	BCP	0.07	0.08	1.95	-	0.21
DHODH	4EF9	4NF	0.23	0.07	3.77	-	0.05
PTR1	5L42	6J6	0.05	0.19	2.50	-	0.36
TR	5EKB	RDS	0.20	0.02	9.66	-	0.49
UGPase	5NZM	9ET	0.29	0.15	2.05	-	0.13

**Table 10:** Error rate and hit rate, calculated for each docking program, by target.

Enzyme	Discarded Molecules	Scoring Functions				
		Moldocksocore	Goldscore	Vina	AD4	ChemPLP
GPDH	8	0	8	8	1	6
DHODH	48	26	10	25	16	20
PTR1	57	16	18	15	2	14
TR	58	21	10	24	4	35
UGPase	35	24	12	1	1	8
E_r_ *		42%	27%	35%	11%	40%
H_r_ *		58%	73%	65%	89%	60%

E_r_ *—Error rate; H_r_ *—Hit rate.

**Table 11:** List of single nucleotide polymorphisms (SNPs) identified in the TritryDB database, with information regarding the ancestral allele, polymorphic allele, allelic frequency, and amino acid position for each species and enzyme studied. The SNPs with the highest allelic frequencies are highlighted in bold.

DHODH					
Species	ID TritryDB	Non-Synonymous SNP	Allele/Amino Acid	Allele Frequency	Protein Position
*L. major*	LMJSD75 16001070	-	-	-	-
*L. braziliensis*	LbrM.16.0550	A	G (Ala)/A (Thr)	0.50/0.50	301
		T	T (Val)/C (Ala)	0.75/0.25	205
		A	G (Gly)/A (Ser)	0.50/0.50	6
**GPDH**					
*L. major*	LmjF.10.0510	C	G (Arg)/C (Pro)	0.83/0.17	180
*L. braziliensis*	LbrM.10.0640	-	-	-	-
**PTR1**					
*L. major*	LmjF.23.0270	A	G (Gly)/A (Glu)	0.60/0.40	25
*L. braziliensis*	LbrM.23.0300	-	-	-	-
**TR**					
*L. major*	LMJLV39 050008400	-	-	-	-
*L. braziliensis*	LbrM.05.0350	T	C (Ala)/T (Val)	0.67/0.33	36
		G	A (Thr)/G (Ala)	0.67/0.33	97
		G	A (Asp)/G (Gly)	0.67/0.33	112
		G	A (Asn)/G (Ser)	0.67/0.33	116
		C	G (Glu)/C (Asp)	0.67/0.33	115
		G	A (Asn)/G (Ser)	0.67/0.33	116
		A	C (Gln)/A (Lys)	0.67/0.33	130
		A	A (Asn)/G (Leu)	0.50/0.50	144
		C	G (Lys)/C (Asn)	0.67/0.33	480
**UGPase**					
*L. major*	LMJLV39 180015400	-	-	-	-
*L. braziliensis*	LbrM.18.1050	-	-	-	-

**Table 12:** Antileishmanial activity of lignans against *L. major* and *L. braziliensis* promastigotes.

ID	Name	IC_50_ (µM)	
		*L. major*	*L. braziliensis*
**156**	Secoisolariciresinol	-	9.28
**158**	Pinoresinol-4-O-β-d-glucopyranoside	>50	36.35
**159**	Epipinoresinol-4-O-β-d-glucopyranoside	36.51	5.39
**160**	Pinoresinol-4-O-β-d-apiofuranosyl-(1→2)-β-d-glucopyranoside	>50	13.77
	Meglumine antimoniate	>40	>40
	Amphotericin B	12.4	18

**Table 13:** Information regarding the selected enzymes deposited in the PDB database and used for docking analysis.

PDB ID	Enzyme	Species	PDB Ligand	Resolution
5NZM	UDP—glucose pyrosphorylase	*L. major*	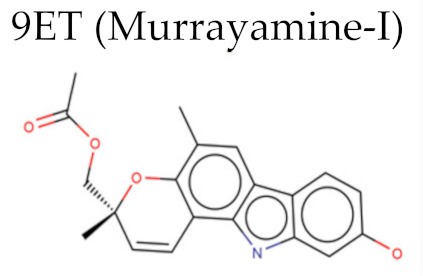	2.35 Å
4EF9	Dihydroorotate dehydrogenase	*L. major*	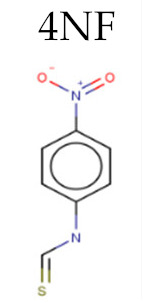	1.6 Å
5L42	Pteridine reductase 1	*L. major*	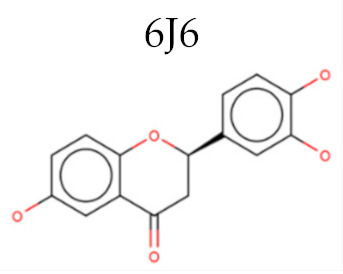	2.1 Å
4WZH	Dihydroorotate dehydrogenase	*L. braziliensis*	-	2.12 Å

## Supplementary Materials

The following are available. Table S1: Lignans and neolignans with good ADMET profiles, Table S2: Predictive assessment of lignin and neolignans toxicity for the evaluated parameters, Figure S1. Alignment of the GPDH protein sequences. The gray regions show non-similar and non-identical amino acids. The red regions show identical amino acids. The yellow regions show similar amino acids. The black boxes indicate the active site, Figure S2: Alignment of the DHODH protein sequences. The gray regions show non-similar and non-identical amino acids. The red regions show identical amino acids. The yellow regions show similar amino acids. The black boxes indicate the active site, Figure S3: Alignment of the PTR1 protein sequences. The gray regions show non-similar and non-identical amino acids. The red regions show identical amino acids. The yellow regions show similar amino acids. The black boxes indicate the active site, Figure S4: Alignment of the TR protein sequences. The gray regions show non-similar and non-identical amino acids. The red regions show identical amino acids. The yellow regions show similar amino acids. The black boxes indicate the active site, Figure S5: Alignment of the UGPase protein sequences. The gray regions show non-similar and non-identical amino acids. The red regions show identical amino acids. The yellow regions show similar amino acids. The black boxes indicate the active site, Table S3: Average of all energy values (EM) obtained from the five scoring functions, for each lignan, and the probability value of potential consensus docking activity (PDC), for each studied enzyme in L. major. Absent values indicate the molecules that were eliminated during this evaluation, Table S4: Average of all energy values (EM) obtained from the five scoring functions, for each lignan, and the probability value of potential consensus docking activity (PDC), for each studied enzyme in L. braziliensis. Absent values indicate the molecules that were eliminated during this evaluation.
