# Supplementary material for: Virtual Screening and the In Vitro Assessment of the Antileishmanial Activity of Lignans
Source: Molecules. 2020 May 12;25(10):2281. doi: 10.3390/molecules25102281 (PMC7288103; doi:10.3390/molecules25102281)
Supplement: Supplementary file 1 [file molecules-25-02281-s001.pdf]

Supplementary Materials

Virtual Screening and the In Vitro Assessment of the Antileishmanial Activity of Lignans

Mayara dos Santos Maia [1], Joanda Paolla Raimundo e Silva [2], Thaís Amanda de Lima Nunes [3], Julyanne Maria Saraiva de Sousa [3], Gabriela Cristina Soares Rodrigues [1], Alex France Messias Monteiro [1], Josean Fechine Tavares [2], Klinger Antonio da Franca Rodrigues [3], Francisco Jaime B. Mendonça-Junior [4], Luciana Scotti [1] and Marcus Tullius Scotti [1,*]

[1] Laboratory of Cheminformatics, Program of Natural and Synthetic Bioactive Products (PgPNSB), Health Sciences Center, Federal University of Paraíba, 58051-900 João Pessoa-PB, Brazil; mayarasmaia@hotmail.com (M.d.S.M.); gaby.ecologia@gnail.com (G.C.S.R.); alexfrancem@ltf.ufpb.br (A.F.M.M.); luciana.scotti@gmail.com (L.S.)

[2] Multi-User Characterization and Analysis Laboratory, Program of Natural and Synthetic Bioactive Products (PgPNSB), Health Sciences Center, Federal University of Paraíba, 58051-900 João Pessoa-PB, Brazil; joanda@ltf.ufpb.br (J.P.R.e.S.); josean@ltf.ufpb.br (J.F.T.)

[3] Infectious Diseases Laboratory, Federal University of Parnaíba Delta, São Benedito, 64202-020 Parnaíba-PI, Brazil; thaisaln13@gmail.com; (T.A.d.L.N.); jully.yanne@gmail.com (J.M.S.d.S.); klinger.antonio@gmail.com (K.A.d.F.R.)

[4] Laboratory of Synthesis and Drug Delivery, State University of Paraíba, 58071-160 João Pessoa-PB, Brazil; franciscojbmendonca@yahoo.com.br

* Correspondence: mtscotti@gmail.com

Table S1. Lignans with good ADMET profiles.

				Pharmacokinetics				
**ID**	**GI[1]**	**BBB[2]**	**P-gp[3]**	**CYP1A2**	**CYP2C19**	**CYP2C9**	**CYP2D6**	**CYP3A4**
**01**	High	No	Yes	No	No	No	No	No
**08**	High	No	Yes	No	No	No	No	No
**20**	High	No	Yes	No	No	No	Yes	No
**30**	High	No	Yes	No	No	No	Yes	No
**31**	High	No	Yes	No	No	No	Yes	No
**32**	High	No	Yes	No	No	No	Yes	No
**44**	High	No	Yes	No	No	No	Yes	No
**57**	High	No	Yes	No	No	No	Yes	No
**58**	High	No	Yes	No	No	No	Yes	No
**59**	High	No	Yes	No	No	No	Yes	No
**60**	High	No	Yes	No	No	No	Yes	No
**61**	High	No	No	No	No	No	Yes	No
**76**	High	No	No	No	No	No	Yes	No
**77**	High	No	No	No	No	No	Yes	No
**83**	High	No	Yes	No	No	No	Yes	No
**84**	High	No	Yes	No	No	No	Yes	No
**86**	High	No	Yes	No	No	No	No	No
**87**	High	No	Yes	No	No	No	Yes	No
**88**	High	No	Yes	No	No	No	No	No
**90**	High	No	Yes	No	No	No	No	No
**91**	High	No	Yes	No	No	No	Yes	No
**121**	High	No	Yes	No	No	No	No	No
**124**	High	No	Yes	No	No	Yes	No	No
**131**	High	No	No	No	No	No	Yes	Yes
**132**	High	No	Yes	No	No	No	No	No
**139**	High	No	Yes	No	No	No	No	No
**140**	High	No	Yes	No	No	No	No	No
**151**	High	No	Yes	No	No	No	Yes	No
**156**	High	No	Yes	No	No	No	Yes	No
**157**	Low	No	Yes	No	No	No	No	No
**158**	Low	No	Yes	No	No	No	No	No
**159**	Low	No	Yes	No	No	No	No	No
**160**	Low	No	Yes	No	No	No	No	No

[1]GI: Gastrointestinal absorption; [2]BBB: Blood–brain barrier; [3]P-gp: P-glycoprotein.

Table S2. Predictive assessment of lignan toxicity for the evaluated parameters.

**ID**	**Mutagenic**	**Tumorigenic**	**Reproductive Effective**	**Irritant**
**1**	No	No	No	No
**8**	No	No	No	No
**20**	No	No	No	No
**30**	No	No	No	No
**31**	No	No	No	No
**32**	No	No	No	No
**44**	No	Low	No	No
**57**	No	No	No	No
**58**	No	No	No	No
**59**	No	No	No	No

**60**	No	No	No	No
**61**	No	No	No	No
**76**	No	No	No	No
**77**	No	No	No	No
**83**	No	No	No	No
**84**	No	No	No	No
**86**	No	No	No	No
**87**	No	No	No	No
**88**	No	No	No	No
**90**	No	No	No	No
**91**	No	No	No	No
**121**	No	No	No	No
**124**	No	No	No	No
**131**	No	No	No	No
**132**	Low	Low	No	No
**139**	No	No	No	No
**140**	No	No	No	No
**151**	No	No	No	No
**156**	No	No	No	No
**157**	No	No	No	No
**158**	No	No	No	No
**159**	No	No	No	No
**160**	No	No	No	No

Table S3. Average of all energy values (EM) obtained from the five scoring functions, for each lignan, and the probability value of potential consensus docking activity (P_{DC}), for each studied enzyme in *L. major*. Absent values indicate the molecules that were eliminated during this evaluation.

ID	GPDH		DHODH		PTR1		TR		UGPase	
	**EM**	**P_{DC}**	**EM**	**P_{DC}**	**EM**	**P_{DC}**	**EM**	**P_{DC}**	**EM**	**P_{DC}**
**44**	58.90	0.82	43.43	0.69	68.81	0.83	63.96	0.91	63.96	0.91
**60**	63.50	0.89	46.47	0.67	67.38	0.82	62.45	0.89	62.45	0.89
**83**	63.94	0.90	59.44	0.67	73.90	0.89	64.63	0.92	64.63	0.92
**86**	63.53	0.89	60.87	0.72	72.16	0.87	63.19	0.90	63.19	0.90
**87**	63.39	0.89	58.81	0.70	-	-	63.95	0.91	63.95	0.91
**124**	57.36	0.64	68.34	0.67	75.47	0.91	69.58	1	69.58	1
**132**	64.43	0.90	49.07	0.66	-	-	62.21	0.89	62.21	0.89
**157**	67.91	0.95	41.51	0.81	72.03	0.87	-	-	50.43	0.72
**158**	66.48	0.93	54.86	0.75	77.00	0.93	-	-	49.97	0.71
**159**	63.43	0.89	53.39	0.87	83.63	1	53.32	0.76	65.55	0.94
**160**	71.03	1	-	0.81	82.16	0.98	53.58	0.77	53.58	0.77
**PDB inhibitor**	37.72	0.53	35.75	0.58	63.94	0.77	37.38	0.53	37.38	0.53

Table S4. Average of all energy values (EM) obtained from the five scoring functions, for each lignan, and the probability value of potential consensus docking activity (P_{DC}), for each studied enzyme in *L. braziliensis*. Absent values indicate the molecules that were eliminated during this evaluation.

ID	GPDH		DHODH		PTR1		TR		UGPase	
	**EM**	**P_{DC}**	**EM**	**P_{DC}**	**EM**	**P_{DC}**	**EM**	**P_{DC}**	**EM**	**P_{DC}**
**8**	59.21	0.86	43.99	0.58	72.53	0.80	-	-	-	-
**20**	58.17	0.85	50.00	0.66	74.64	0.83	56.19	0.76	-	-
**31**	60.39	0.88	48.93	0.65	78.41	0.87	57.66	0.78	62	0.77

**32**	59.15	0.86	48.32	0.64	78.54	0.87	55.41	0.75	63	0.78
**44**	45.54	0.66	48.07	0.64	82.79	0.92	55.57	0.75	64	0.80
**57**	55.88	0.81	47.32	0.63	80.91	0.90	54.14	0.73	64	0.80
**58**	50.37	0.73	46.09	0.61	68.36	0.76	53.10	0.72	64	0.80
**59**	49.23	0.72	44.79	0.59	70.40	0.78	-	-	64	0.80
**60**	56.97	0.83	46.11	0.61	77.81	0.86	54.06	0.73	66	0.83
**61**	56.56	0.82	52.94	0.70	-	-	55.24	0.75	-	-
**76**	-	-	44.90	0.59	-	-	-	-	-	-
**83**	59.50	0.87	54.88	0.73	77.25	0.86	64.98	0.80	61	0.77
**87**	60.87	0.89	49.12	0.65	71.98	0.80	59.44	0.80	62	0.78
**91**	61.54	0.90	48.37	0.64	78.16	0.87	57.35	0.77	61	0.77
**121**	61.30	0.89	47.32	0.63	76.26	0.85	-	-	-	-
**124**	56.49	0.82	51.88	0.69	81.29	0.90	65.78	0.89	-	-
**156**	53.24	0.78	51.39	0.68	74.87	0.83	54.11	0.73	66	0.83
**157**	58.25	0.85	63.68	0.84	89.59	1	60.59	0.82	66	0.83
**158**	68.18	1	55.11	0.73	77.40	0.86	62.11	0.84	68	0.85
**159**	68.07	0.99	55.19	0.73	82.16	0.91	73.53	1	79	1
**160**	57.54	0.84	74.97	1	79.52	0.88	-	-	76	0.95
**PDB inhibitor**	36.26	0.53	32.40	0.43	67.31	0.75	53.15	0.72	61	0.76

```
L. major          MISTKRHINTNELLHLSKAVVFGSGAFGTALAMVLSKKCREVCVWHIKEEEARLVNEKRE 60
L. braziliensis   MLSANGKANANELLYLKKAVVFGSGAFGTALATVLAKKCREVCVWHIKEEEAQLVNRKRE 60

L. major          NDLYLRGVQLASNIIFTSDVDEAYKGAELILFVIPTQFLRGFFQKSGGNLIAYAKARQVP 120
L. braziliensis   NMMYLKGVKLASNITFTSNVEQAYKDAEIILFVIPTQFLRGFFQKSGGNLITYAKEKRVP 120

L. major          VLVCTKGIERSTLKFPAQIVGEFFPSNLLSVLAGPSFAIEVATGVFTCVSVASADINVAR 180
L. braziliensis   VLVCTKGIERSTLKFPAQIVGEFFADYPLSVLAGPSFAIEVAAGIFTCVGIASADINQAR 180

L. major          RLQRIMTTGDRSFVCWATTDTVGCEVASAVKNVLAIGSGVANGLGMGLNARAALITRGLL 240
L. braziliensis   RLQRIMTTGDRTFVCWATTDTIGFEVASAVKNVLAIGSGVANGLGMGFNARAALITRGLL 240

L. major          EIRDLTAALGGDGSAIFGLAGFGDLQLTCSSELSRNFTVGKKLGKGLSLEEIQRTSKAVA 300
L. braziliensis   EIRDLTGALGGDGSAVFGLAGLGDLQLTCSSELSRNFTVGKRLGEGTSIEEIQRTSKAVA 300

L. major          EGVATAEPLVRLAQQLKVTMPLCQQIYEVVYKKKNPRAAIADLLSCGLQDEGLPPLFKKS 360
L. braziliensis   EGVATAEPLMRLAQQLKVSMPLCKEIYKIVYEKKNPRDAIADLLSCGLQDEGLPALFKKS 360

L. major          AATPSKL 367
L. braziliensis   SATLSKL 367
```

Figure S1. Alignment of the GPDH protein sequences. The gray regions show non-similar and non-identical amino acids. The red regions show identical amino acids. The yellow regions show similar amino acids. The black boxes indicate the active site.

```
L. major          MGSSHHHHHHSSGLVPAGSHMASMTGGQQMGRGSMSLQVNLLNNTFANPFMNAAGVMCTT 60
L. braziliensis   MGSSHHHHHHSSGLVPRGSHMASMTGGGQMGRGSMSLQVGILGNTFANPFMNAAGVMCST 60

L. major          TEELVAMTESASGSLVSKSCTPALREGNPTPRYQALPLGSINSMGLPNNGFDFYLAYAAE 120
L. braziliensis   EEELAAMTESTSGSLITKSCTPALREGNPAPRYYTLPLGSINSMGLPNKGFDFYLAYSAR 120

L. major          QHDYGKKPLFLSMSGLSMRENVEMCKRLAAVATEKGVILELNLSCPNVPGKPQVAYDFDA 180
L. braziliensis   HHDYSRKPLFISISGFSAEENAEMCKRLAPVAAEKGVILELNLSCPNVPGKPQVAYDFDA 180

L. major          MRQCLTAVSEVYPHSFGVKMPPYFDFAHFDAAAEILNEFPKVQFITCINSIGNGLVIDAE 240
L. braziliensis   MRRYLAAISEAYPHPFGVKMPPYFDFAHFDAAAEILNQFPKVQFITCINSIGNGLVIDVE 240

L. major          TESVVIKPKQGFGGLGGRYVLPTALANINAFYRRCPGKLIFGCGGVYTGEDAFLHVLAGA 300
L. braziliensis   TESVVIKPKQGFGGLGGRYVFPTALANVNAFYRRCPGKLIFGCGGVYTGEDAFLHVLAGA 300

L. major          SMVQVGTALQEEGPSIFERLTSELLGVMAKKRYQTLDEFRGKVRTLDGTAESTR       354
L. braziliensis   SMVQVGTALHEEGAAIFERLTAELLDVMAKKGYKALDEFRGKVKAMD-------       347
```

Figure 2. Alignment of the DHODH protein sequences. The gray regions show non-similar and non-identical amino acids. The red regions show identical amino acids. The yellow regions show similar amino acids. The black boxes indicate the active site.

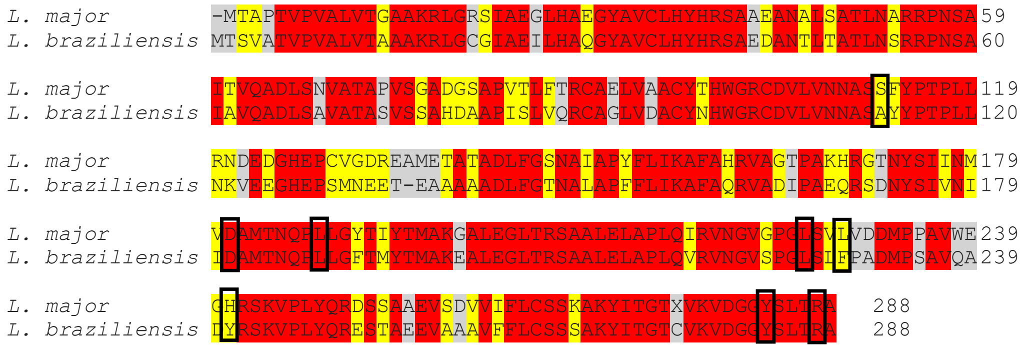

Figure 3. Alignment of the PTR1 protein sequences. The gray regions show non-similar and non-identical amino acids. The red regions show identical amino acids. The yellow regions show similar amino acids. The black boxes indicate the active site.

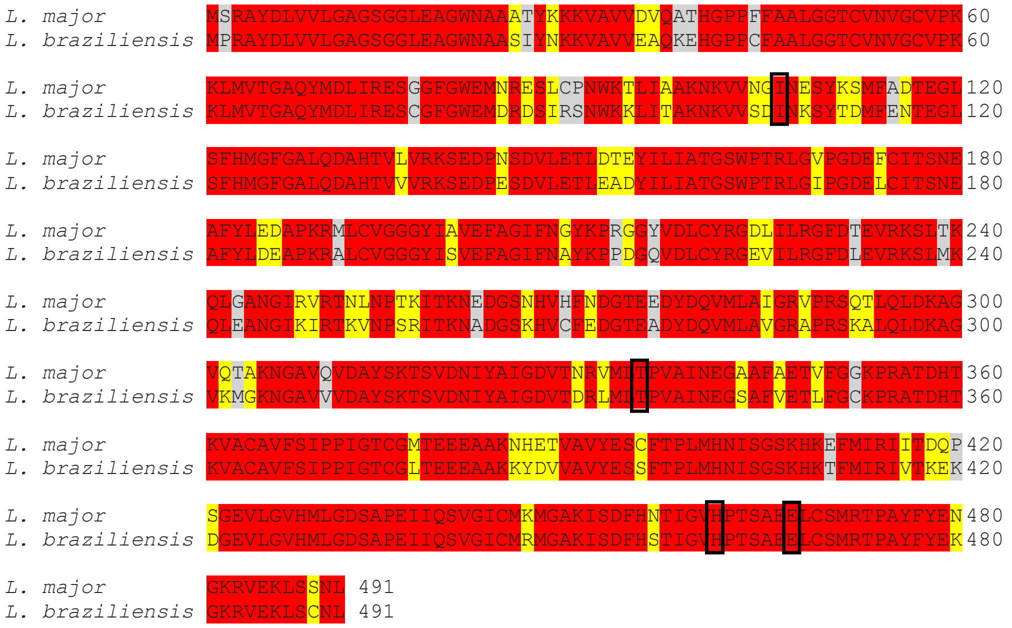

Figure S4. Alignment of the TR protein sequences. The gray regions show non-similar and non-identical amino acids. The red regions show identical amino acids. The yellow regions show similar amino acids. The black boxes indicate the active site.

```
L. major          MENDMKSLSAAAQACVKKMRDAKVNEACIRTFIAQHVMVSKGETGSIPDSAIMPVDSLDA 60
L. braziliensis   MEIDMKAISAAAQACVKKMRDAKVNEACIRTFIAQHVMVSNGETGSIPDSAITRIESLDE 60

L. major          LDSLTIECDNAVLQSTVVLKLNGGLGTGMGLCDAKTLLEVKDGKTFLDFTALQVQYLRQH 120
L. braziliensis   LEGLTTECDNAVLQSTVVLKLNGGLGTGMGLHDAKTLLDVKDGKTFLDFTALQVQYLRQH 120

L. major          CSEHLRFMLMDSFNTSASTKSFLKARYPWLYQVFDSEVELMQNQVPKILQDTLEPAAWAE 180
L. braziliensis   CSERLRFMLMNSFNTSASTRRFLEARYPWLYQVFDSEVELMQNQVPKILQDTLEPVTWPE 180

L. major          NPAYEWAPPGHGDIYTALYGSGKLQELVEQGYRYMFVSNGDNLGATIDKRVLAYMEKEKI 240
L. braziliensis   DPGCEWAPPGHGDIYTALYGSGKLQELVNQGYRYIFVSNGDNLGATIDKRVLAYMEEKQI 240

L. major          DFLMEVCRRTESDKKGGHLARQTVYVKGKDGQPDAEK-RVLLLRESAQCPKADMESFQDI 299
L. braziliensis   DFLMEVCRRTESDKKGGHLARQVVCAKSKGSQPDASTGGLLLLRELAQCPKEDMNNFQDI 300

L. major          NKYSFFNTNNLWIRLPVLLETMQEHGGTLPLPVIRNEKTVDSSNSASPKVYQLETAMGAA 359
L. braziliensis   NKHSFFNTNNLWIRLPALLATMEKHGGTLPLPVIRNEKTVDPSNPASPKVYQLETAMGAA 360

L. major          IAMFESASAIVVPRSRFAPVKTCADLLALRSDAYVVTDDFRLVLDDRCHGHPPVVDLDSA 419
L. braziliensis   IAMFENASALVVPRSRFAPVKTCADLLALRSDAYVVTNDSRLVLDDRCHGHPPVVDLDNA 420

L. major          HYKMMNGFEKLVQHGVPSLVECKRVTVKGLVQFGAGNVLTGTVTIENTDSASAFVIPDGA 479
L. braziliensis   HYKMMGGFEKLVQNGVPSLVKCKRLIVKGLVQFGTDNVLTGTVKIENAHSASAFVTPDGA 480

L. major          KLNDTTASPQQSTNKMRPLEHHHHHH 505
L. braziliensis   KLTDTTVSPP---------------- 490
```

Figure S5. Alignment of the UGPase protein sequences. The gray regions show non-similar and non-identical amino acids. The red regions show identical amino acids. The yellow regions show similar amino acids. The black boxes indicate the active site.
